# Sleep slow oscillation emergence on the scalp as a renewal point process

**DOI:** 10.1371/journal.pcbi.1014572

**Published:** 2026-07-29

**Authors:** Mahmoud Alipour, Sara C. Mednick, Paola Malerba

**Affiliations:** 1 Center for Biobehavioral Health, Abigail Wexner Research Institute, Nationwide Children’s Hospital, Columbus, Ohio, United States of America; 2 Department of Pediatrics, The Ohio State University, College of Medicine, Columbus, Ohio, United States of America; 3 Department of Cognitive Sciences, University of California Irvine, Irvine, California, United States of America; Imperial College London, UNITED KINGDOM OF GREAT BRITAIN AND NORTHERN IRELAND

## Abstract

Sleep slow oscillations (SOs), characteristic of NREM sleep, are causally tied to cognitive outcomes and the health-promoting homeostatic functions of sleep. Characterization of SO organization during a night of sleep is an active area of research, with most existing work focused on individual SO events rather than the temporal dynamics across sleep cycles or channels. Hence, the probabilistic structure governing the timing and distribution of SOs in one individual across the sleep night remains underexplored. To address this gap, we introduce a computational model characterizing SO emergence over time as a function of sleep cycle and electrode location. SOs were detected in a dataset of nighttime sleep from 22 subjects (9 females), acquired with polysomnography including 64 EEG channels. Modeling of SO occurrence was performed separately for SOs detected during stage N3, and during a combination of stages N2 and N3 (N2&N3). We analyzed SO emergence at two temporal scales. First, we modeled cumulative SO occurrences across successive sleep cycles using a power law fit (across-cycles model). Second, we characterized SO timing within each cycle using a renewal point process (within-cycle model), fitting an inverse Gaussian distribution to the inter-event intervals of SOs and estimating its parameters μ (mean) and λ (shape) for each sleep cycle and channel. Both models were fit to individuals and to a generic idealized ‘average’ SO emergence behavior, describing both general and individualized patterns. The decay rate of SO count per cycle was 1.70 for N3 and 1.14 for N2&N3, with participant-level variance of 1.00 and 0.53, respectively. Within-cycle modeling showed consistent increases in μ (0.83 ± 0.14) and λ (4.59 ± 0.66) across cycles. This probabilistic framework captures structured SO timing and supports descriptive modeling of large-scale SO dynamics across the night, offering a basis for future investigations of variability in sleep organization.

## Author summary

During sleep, the brain generates rhythmic electrical waves called slow oscillations (SOs) that are critical for memory consolidation, brain waste clearance, and cognitive health. While decades of research have examined individual SO events, how these oscillations are organized across an entire night of sleep has remained largely unknown. We developed a mathematical framework to characterize SO timing at two scales: across successive sleep cycles, and within each individual cycle. Using overnight EEG recordings from 22 healthy adults, we show that SO occurrence follows structured patterns rather than being purely random. Across the night, SOs become progressively less frequent in a manner well described by a power law. Within each sleep cycle, the timing between successive SOs can be captured by a renewal point process model that reveals increasing regularity as the night advances. Importantly, these patterns can be characterized from early sleep cycles and used to describe later dynamics. This framework provides a new way to understand how the brain organizes its slow-wave activity over the course of sleep. Beyond basic science, it may help improve closed-loop brain stimulation technologies—which aim to enhance memory and sleep quality—by enabling better prediction of when the next slow oscillation will occur.

## 1. Introduction

Cortical slow oscillations (SOs, 0.5-1.5 Hz) have been experimentally linked to health and cognitive functions of sleep such as synaptic homeostasis [1,2], glymphatic clearance [3], and memory consolidation [4,5]. The emergence of individual SO events has been well characterized from an event-level perspective, focusing on circuit-level dynamics around the SO trough—particularly the transition from down-state to up-state—and corresponding changes in firing rates [6–8]. At the other end of the spectrum, large-scale mean field models simulate average SO dynamics in EEG recordings but typically overlook the temporal organization of discrete SO events across the night [9,10]. As a result, the question of how SOs are structured in time across a night—both within and across sleep cycles—remains largely unanswered. Given that SOs contribute to slow-wave activity (SWA) [11], which declines systematically across successive sleep cycles [12], their emergence is unlikely to be purely random. We hypothesize that SOs arise from a combination of structured and stochastic processes that unfold over time and across cortical regions. Understanding this probabilistic organization is essential for identifying features that generalize across individuals versus those that are specific to a given person or night.

This perspective is particularly relevant for studies aiming to enhance SOs through closed-loop stimulation, which typically rely on real-time detection at single electrodes but overlook broader temporal dynamics [13,14]. Although experimental studies have demonstrated that electrical or auditory stimulation can enhance SOs [14–17], outcomes across studies and populations have been inconsistent [18–21]. One likely contributor to this variability is the stochastic nature of SO emergence — both in timing and spatial distribution across the scalp — which complicates reliable detection and interpretation. While these limitations have motivated efforts to improve stimulation protocols, they also point to a more fundamental need: to better understand the probabilistic architecture underlying SO emergence. The present work proposes descriptive model of SO timing, offering a powerful framework for understanding the large-scale organization of spontaneous sleep and its variability across individuals.

In this study, we introduce a computational modeling framework that characterizes the temporal structure of SOs across the night. Using full-night EEG recordings from 22 participants, we modeled SO occurrence along two temporal dimensions: across successive sleep cycles and within individual cycles. A power-law model was used to capture the decline in SO count across cycles, while a renewal point process—implemented with an inverse Gaussian distribution—was used to model inter-event intervals within each cycle. Parameters were estimated for each electrode and subject, allowing us to examine both generic patterns and individualized trends. This approach reveals structured, probabilistic dynamics of SO emergence that can serve as a foundation for future studies of individual variability, cognitive function, and clinical alterations in sleep architecture.

## 2. Materials and methods

### 2.1. The sleep EEG dataset

The present study utilizes a dataset of sleep polysomnography from the Sleep and Cognition Lab at University of California Irvine, led by Dr. Mednick. The dataset is introduced in detail in [22]. The dataset includes full-night sleep EEG data from 22 healthy volunteers (9 females, age 18–34 years) without psychological or neurological issues. EEG were recorded using a 64-channel cap placed according to the international 10–20 System at a 1,000 Hz sampling rate, which was subsequently down-sampled to 128 Hz. Out of the 64 channels, 56 recorded the electrical activity of the brain, while others served as reference, ground, and other biosignal channels (e.g., EOG, EMG). In our analysis, EEG signals were re-referenced to the contralateral mastoids (M1 and M2 channels). Sleep stages (Wake, N1, N2, N3, and REM sleep) were visually scored in 30-second epochs following the R&K manual [23], using the MATLAB toolbox HUME [24]. R&K stages 3 and 4 were combined into a single stage (N3) in accordance with current AASM nomenclature [25], a practice widely used in the literature [26,27]. This study relies on manually sleep-staged data for analysis. Participants all had a good quality sleep (basic sleep outcomes in S1 Table). The dataset used in this study consists solely of spontaneous sleep recordings, and does not include any experimental interventions.

### 2.2. SO detection

SO detection was performed at each electrode independently, using an algorithm previously utilized in [22,28], closely following the criteria introduced by Massimini et al. [29] and Dang-Vu et al. [30]. The algorithm was applied only to artifact-free data segments, as described below.

#### 2.2.1. Artifact removal.

EEG epochs containing two types of artifacts were excluded. First, 30-second epochs containing artifacts such as movements or arousals, as determined by the expert sleep scorer at the Sleep and Cognition Lab at the University of California, Irvine, were discarded. Second, epochs with muscle movement artifacts were algorithmically identified and excluded using two methods. Following Brunner and colleagues [31], we identified as artifacts 4-second-long time bins in which power in the 26.25–32 Hz range exceeded 4 times the median of the 45 surrounding bins (3 minutes around the epoch). Similarly, following Wang and colleagues [32], we identified as artifacts 5-second-long time bins in which power in the 4–50 Hz range exceeded 6 times the median of all bins.

#### 2.2.2. Signal filtering.

The EEG referenced to contralateral mastoids was bandpass filtered between 0.1–4 Hz using a 4th-order Butterworth filter applied in forward and reverse directions (MATLAB’s filtfilt function). This zero-phase filtering implementation resulted in an effective 8th-order filter with 24 dB attenuation at the cutoff frequencies. This approach ensures no phase distortion, which is crucial for preserving the temporal characteristics of slow oscillations.

#### 2.2.3. SO detection criteria.

Potential SOs were identified in artifact-free epochs of NREM sleep by analyzing each channel independently. Candidate SOs were segments between consecutive positive-to-negative and negative-to-positive transitions that satisfied the following criteria: (1) trough amplitude ≤ −80 μV, (2) peak-to-peak voltage range ≥ 80 μV, (3) the time between the first and second zero crossing (i.e., the duration of the negative half-wave) fell within 300–1000 ms, and (4) total event duration ≤ 10 seconds. The peak-to-peak threshold of 80 μV was determined empirically based on the amplitude distribution of candidate events in this dataset — thresholds reported in the SO literature vary across studies (e.g., 75, or 140 μV), reflecting differences in recording setups and populations [29,33,34]. Given the waveform structure around zero-crossings, criterion (1) implies a peak-to-peak range of at least 80 μV in these data, accounting for the numerical equivalence of criteria (1) and (2). Both are nevertheless retained explicitly for transparency and consistency with standard SO reporting conventions [30,35]. A comprehensive characterization of detected SO properties — including duration, trough amplitude, and before- and after-trough slopes measures across sleep stages (S2 Table; S1 and S2 Figs), sleep cycles (S3 Table; S3 Fig), and anterior-to-posterior electrode gradients (Frontal, Central, Parietal, Occipital groups; S4–S6 Figs) — is provided to confirm the physiological consistency and spatial homogeneity of detected events, consistent with established SO detection approaches in the literature [28–30,36].

#### 2.2.4. Amplitude-based selection of candidate SO events.

The pool of candidate SO events that met these parameters underwent further screening to eliminate potential artifacts. This involved computing the amplitude at the trough referenced to the average signal ±10 seconds around the minimum. Events at one electrode with an amplitude size 4 standard deviations above the mean of all events detected at that electrode were discarded. A secondary distribution of amplitudes, encompassing all events from all electrodes of a subject, was then created. Once again, events with amplitudes above 4 standard deviations from the mean were discarded. When selecting SOs occurring during a stage, only those with both the beginning and end falling within the sleep stage were considered. EEG signals were referenced to the contralateral mastoids, thereby minimizing the influence of global signal redistribution across electrodes. Slow oscillation detection and amplitude-based screening were performed independently at each electrode using local waveform features. The two-stage screening procedure—first at the electrode level and then across electrodes within each subject—was designed to remove amplitude outliers while avoiding bias from region-specific large-amplitude events.

### 2.3. Sleep cycle detection

For sleep cycle detection, we utilized the Feinberg and Floyd algorithm [37]. Briefly, a sleep cycle is defined as the interval from the onset of stage N2 in one NREM period to the onset of stage N2 in the next NREM period, encompassing the intervening REM episode. This algorithm requires a minimum of 5 minutes for the REM period — except for the first REM episode, for which no minimum duration is required — and at least 15 minutes for the NREM period, to prevent brief stage N2 epochs in the REM period from being considered as separate NREM periods. Additionally, the start of a cycle is determined by the start of stage 2. In our study, we consider only sleep cycles that include both REM and NREM periods. Therefore, we excluded the last sleep cycle of participants if they showed no REM period.

### 2.4. SO percentage measurement

This study investigates patterns in SO counts across successive cycles. To provide a comparable measure across participants, we use SO percentage, a normalized value for each participant. We measure the SO percentage during successive EEG epochs and sleep cycles using the following formulas:


$$\begin{array}{c} SO\;percentage\;in\;the\;epoch=\frac{SO\;count\;in\;the\;epoch}{Total\;SO\;count\;in\;the\;nighttime\;sleep}\times \text{1}\text{0}\text{0}\; \end{array}$$ (1)

$$\begin{array}{c} SO\;percentage\;in\;the\;cycle=\frac{SO\;count\;in\;the\;cycle}{Total\;SO\;count\;in\;the\;nighttime\;sleep}\times \text{1}\text{0}\text{0}\; \end{array}$$ (2)

SO counts used in these formulas were pooled across all 56 EEG channels, such that each SO event detected at any electrode within a given epoch or cycle contributed to the count. This all-channel aggregation was adopted to capture the overall temporal SO activity across the night. This study focuses on SO percentage during stage N3 and the combined stages N2 and N3 (N2&N3) of the NREM period. We measure SO percentage in 30-second epochs, which we encode as a time series of SO percentage in successive epochs. As we were interested in investigating the pattern of SO percentage across successive cycles and participants, we noted the variability in sleep stages counts in sleep cycles and the resulting different lengths of NREM periods. To achieve comparability, we scaled the length of each NREM period to a fixed 100-point time series. S7 Fig displays the average epoch count across participants in the first four sleep cycles in our dataset. In each NREM period, we utilize a method adapted from our previous work [38] to conduct a temporal normalization. Specifically, the SO percentage of epochs was first calculated using Relationship 1 to yield a time series for each cycle. Because NREM periods vary in duration, these series have a variable number of points (L). Each series was up-sampled via linear interpolation onto a high-resolution grid (e.g., 100 × L points) and subsequently down-sampled by a factor of L. This procedure results in a standardized 100-point time series for every cycle, effectively normalizing out differences in NREM duration.

### 2.5. Modeling of SO dynamics across sleep cycles

To characterize the evolution of SO occurrence across the night, we modeled the change in SO percentage across successive sleep cycles using parametric functions describing across-cycle decay. We considered two candidate models: a power-law formulation and an exponential formulation. The primary model was a power-law function of the form:


$$\begin{array}{c} y(C)=x_{\text{0}}.C^{T}\; \end{array}$$ (3)

where y(C) represents the SO percentage in cycle C, $x_{\text{0}}\;$is the SO percentage in the first cycle, and T is the decay exponent. As an alternative, we evaluated an anchored exponential model of the form:


$$\begin{array}{c} y(C)=x_{\text{0}}.e^{\frac{C-\text{1}}{R}}\; \end{array}$$ (4)

where b is the decay parameter. In both models, $x_{\text{0}}$ was fixed to the first cycle value, such that only a single parameter (T or b) was estimated. Model fitting was performed at both the group level and the individual subject level using least-squares estimation. Model performance was evaluated using goodness-of-fit metrics including the coefficient of determination (R^2^) and root mean squared error (RMSE).

### 2.6. Modeling of inter-arrival time of SOs

This study models the timing of SO occurrence within and across cycles. First, we describe our modeling approach for the timing of SO occurrence within cycles. Then, by modeling the parameters of the model across cycles, we introduce a probabilistic modeling of SO occurrence using a point process [39]. Point process models are commonly used in neuroscience to describe the statistical structure of temporally discrete events, such as neuronal spikes or oscillatory bursts [40]. The first step in modeling such events involves identifying a probability distribution function (PDF) that fits the empirical inter-event intervals. After determining the PDF for event occurrence, the inversion method, which is a commonly used technique for simulating random variates, can be employed to simulate event occurrences. The inversion method samples from a uniform distribution and then uses the inverse of the cumulative distribution function (CDF) to transform these uniform samples into samples from the desired distribution [41]. Since the CDF describes the probability that a random variable takes on a value less than or equal to a specified value [42], the inverse CDF describes the value of the random variable corresponding to a given probability. By generating random samples from a uniform distribution for the inverse CDF, we can simulate the occurrence of events ^41^.

This study employed an inverse Gaussian (IG) function as a PDF to model the probability distribution of SO occurrence inter-arrival times. Among the candidate distributions evaluated (S4 Table), the IG showed the highest overall similarity across sleep cycles when averaged across the dataset. Notably, in the first sleep cycle—whose parameter estimates were used to extrapolate model behavior across subsequent cycles—the IG provided a better fit than the others. In addition to this empirical performance, the IG distribution is well suited for modeling inter-arrival times of discrete events because it is defined only for positive values and captures the skewed structure commonly observed in neural event interval distributions [43]. Furthermore, the IG distribution has been widely used in neuroscience to model stochastic neural timing processes such as spike trains and event intervals [44–46]. For these reasons, we selected the IG distribution to model SO inter-arrival times. The IG model consists of two parameters: mean (μ) and lambda (λ), defined according to the following formula [47]:


$$\begin{array}{c} f(x;\mu ,\;\lambda )=\sqrt{\frac{\lambda }{\text{2}\pi x^{\text{3}}}}\;exp\left(-\frac{\lambda (x-\mu {)}^{\text{2}}}{\text{2}\mu^{\text{2}}x}\right)\; \end{array}$$ (5)

where x is the random variable. The optimal parameters of the IG model were determined by fitting an IG model to the distribution of the inter-arrival times of SOs within each sleep cycle. The CDF of the IG can be described by the following formula [48]:


$$\begin{array}{c} F(x)=\emptyset \left[\sqrt{\frac{\lambda }{x}}\left(\frac{x}{\mu }-\text{1}\right)\right]+e^{\left(\frac{\text{2}\lambda }{\mu }\right)\emptyset \left[-\sqrt{\frac{\lambda }{x}}\left(\frac{x}{\mu }+\text{1}\right)\right]}\; \end{array}$$ (6)

where ∅ is CDF of the standard normal distribution, as described by the formula below [49]:


$$\begin{array}{c} \emptyset (t)=\frac{\text{1}}{\sqrt{\text{2}\pi }}\int_{-\infty }^{x}e^{-\frac{t^{\text{2}}}{\text{2}}}\;dt\; \end{array}$$ (7)

The CDF of normal distribution and its inverse are not available in closed form, requiring the use of numerical procedures [50]. Consequently, the inverse CDF of IG is also not available in a closed form. Therefore, we employed MATLAB to estimate the inverse CDF of the IG distribution through numerical procedures, leveraging the error function [51] for estimating inverse CDF [52].

By fitting the IG model to the inter-arrival times of SOs within each sleep cycle, we obtained μ and λ parameters for every cycle. Parameter estimation was performed independently for each cycle, at each electrode and for each subject, resulting in four independent sets of cycle-specific parameter estimates. In a separate analysis, parameters derived from the first sleep cycle were used to simulate SO timing in Cycles 2–4, enabling evaluation of the extent to which early-night dynamics predict later-cycle behavior. This process was repeated independently across all 56 EEG channels for each of the 22 participants, resulting in a 56-by-22 matrix of μ and λ values per cycle. That is, SO inter-arrival times were computed from the trough timestamps of SOs detected at each individual electrode separately, and the IG model was fitted to each channel’s time series independently. The reported μ and λ values therefore reflect averages of these channel-level parameter estimates across subjects and electrodes. Parameter estimation was performed independently for each electrode and each participant using the empirical inter-arrival times at that specific channel-subject-cycle combination. The reported group-level values therefore represent averages of individual channel × subject estimates, computed separately per cycle, rather than estimates derived from a pooled or averaged time course. To characterize how SO timing dynamics evolve across the night, we analyzed the variation of these parameters across successive cycles. Specifically, we examined whether μ and λ values estimated from early cycles could be used to describe their progression in later cycles. For each cycle, we averaged μ and λ across electrodes and participants to obtain representative values ($\overset{―}{\mu }$ and $\overset{―}{\lambda }$). The functional forms used to describe the cross-cycle evolution of μ and λ were selected empirically on the basis of goodness-of-fit and biophysical interpretability. For μ, a power-law model was chosen because the trajectory was well-linearized in log–log space, and because this form is consistent with the power-law model applied to SO counts across cycles (Section 2.5), providing a unified modeling framework. For λ, the trajectory was better linearized in log-linear space, motivating an exponential function analogous to the anchored exponential model in Eq. 4. Both models are anchored to first-cycle estimates, so that later-cycle behavior is predicted from early-night dynamics. Prior to averaging, sleep cycles with a low SO count (below a cycle-specific threshold) were excluded, where the threshold was defined as the median of the lower quartile of SO counts across channels and subjects. Eight electrodes (TP7, TP8, P1, P2, P5, P6, PO3, and PO4) were excluded entirely from this modeling due to insufficient SO counts. For modeling the inter-arrival time of SO occurrence, we focused exclusively on N3.

To simulate the inter-arrival time of SOs, we generated random numbers from a uniform distribution. We then used the inverse CDF to convert those values to simulated inter-arrival times of SOs. Only inter-arrival times exceeding a minimum inter-event interval for the given sleep cycle and electrode were accepted into the simulation. This minimum inter-event interval was defined empirically as the smallest observed inter-trough interval at each electrode for each subject and sleep cycle — that is, the minimum value of the pairwise differences between successive SO trough timestamps. For use in the simulation, this value was averaged across subjects per electrode and cycle. This threshold serves as a data-driven lower bound on the inter-arrival time distribution, ensuring that simulated SO sequences respect the minimum spacing observed in the recorded data. Physiologically, it approximates the minimum time required for the thalamocortical network to complete one SO event before a new SO trough can be detected, and therefore reflects the data-derived time duration in between two subsequent SO troughs. The simulation was built on an iterative process, including continued generation of simulated inter-arrival time values until the last inter-arrival time exceeded the cumulative summation of all inter-arrival times of SOs.

### 2.7. Model evaluation

The primary goal of this study is to model the timing of SO events using probabilistic distributions, rather than to classify individual events or generate pointwise predictions. Accordingly, we evaluated model performance using goodness-of-fit metrics that quantify how well the simulated inter-arrival distributions match observed data. This approach emphasizes probabilistic structure over detection accuracy, and avoids reliance on sensitivity or specificity, which are suited to classification tasks.

We used three primary metrics to quantify model fit: absolute difference error, the Kolmogorov–Smirnov (KS) test, and Kullback–Leibler (KL) divergence, each applied using 100 bootstrapped simulations per cycle. Absolute difference error measures the average relative deviation between model-generated and empirical SO timings:


$$\begin{array}{c} Absolute\;difference\;error=\frac{\text{1}}{\text{1}\text{0}\text{0}}\sum |\frac{\left(prediction-data\right)}{data}|\; \end{array}$$ (8)

KL divergence is a measure to quantify the dissimilarity between two probability distributions by explaining how one probability distribution diverges from another [53]. It can be defined using the following formula:


$$\begin{array}{c} D_{KL}(P||Q)=\;\sum_{x\epsilon \chi }p(x)\;log\left(\frac{p(x)}{q(x)}\right)\; \end{array}$$ (9)

where P and Q are probability distributions on the same sample space 𝜒, and x is the bin number in the histogram. p(x) and q(x) are probability density function of P and Q respectively. Values of this measure closer to zero indicate lower dissimilarity.

We used the KS-test to assess whether the distributions of our data and simulations could be derived from the same probability distribution. To obtain a cumulative value across multiple simulations, we defined a “distribution similarity” metric, as the fraction of 100 simulations which passed the KS-test (p-value > 0.05) when compared to a sample of real data. We interpret this measure as an indicator of similarity, ranging between 0 and 1, with values closer to 1 indicating higher similarity. It should be noted that, as a statistic derived from the KS test, this metric is sensitive to the number of inter-arrival intervals available in each cycle. Because SO counts decline across successive cycles, the KS test has lower statistical power to detect distributional differences in later cycles, which introduces a sample-size-dependent component when comparing distribution similarity values across cycles. For this reason, this metric should be interpreted alongside the absolute difference error and KL divergence, which provide complementary and more sample-size-stable assessments of distributional correspondence.

In addition to the three mentioned measures, we utilize a probability-probability (P-P) plot to visually assess the performance of the model by examining how closely a dataset aligns with a particular model. This method entails plotting the two CDFs against each other. If they closely resemble each other, the data will appear as a nearly straight line [54]. Confidence intervals represent the lower and upper bounds of the range within which we expect the CDF of the data to lie with a certain level of confidence, 95% in this study. If the points in the P-P plot closely adhere to the diagonal line (y = x), it indicates a good fit between the distribution of the data and the model. Conversely, if a significant number of points in the P-P plot fall outside the confidence intervals, it may suggest a lack of fit between the distributions of the data and the model.

To represent the performance of the model in a P-P plot across all subjects, we followed the method outlined in reference [38] to standardize the CDF lengths of both the data and model to 250 points for each participant. In this method for standardizing the lengths of different signals, each signal is first interpolated to a fixed length N (in this study, 250 points) by generating additional data points between successive samples using a uniform interpolation method. The interpolated signal is then down-sampled at a rate proportional to its original length, resulting in a final signal scaled to the desired length N. Subsequently, we computed the mean CDF across all participants for both the data and the model. Plotting these mean CDFs against each other for each cycle produced the P-P plot. This resulting plot visually illustrates the average performance of the model across all participants in our study.

This study employs evaluation methods to assess how well the model replicates the empirical distribution of SO inter-arrival times. We use quantitative metrics—including absolute difference error, KL divergence, and the KS test—alongside visual tools such as P–P plots to evaluate the alignment between model-generated and observed data. These comparisons help characterize how accurately the model captures the probabilistic structure of SO timing across the night.

## 3. Results

We modeled SO occurrence across the night in two stages: first, by analyzing trends in SO percentage across successive sleep cycles, and second, by characterizing the timing of SOs within each cycle using inter-arrival distributions. This ordering reflects a coarse-to-fine approach, in which we first establish the general temporal organization of SO activity across the night, and then examine the finer-scale probabilistic structure governing event timing within individual cycles. In both steps, model parameters were derived from information obtained during the first sleep cycle. This approach enabled us to examine whether early night SO dynamics could describe or account for temporal structure observed in later cycles.

### 3.1. SO percentage across cycles

**Fig 1:**
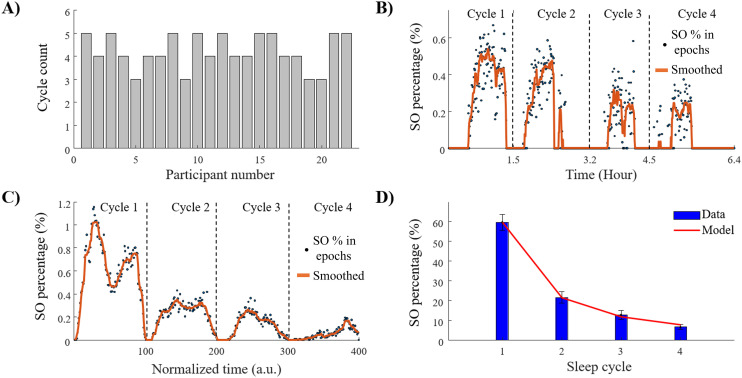
SO percentage across successive cycles. A) cycle count in different participants of our dataset. The x-axis shows participant number in the dataset. Most participants have 4 or fewer cycle. The average cycle count in our study is 4.2. **B)** SO percentage in successive epochs across cycles during N3 of the first participant in our dataset. The x-axis shows time per hour from sleep onset to end of fourth cycle. **C)** SO percentage across successive cycles during N3 by averaging across all participants. The x-axis shows normalized time, with cycle length equalized to 100 points. **D)** Total SO percentage during N3 across successive cycles obtained by averaging SO percentage across all participants. The across-cycle trend was modeled using a power-law function $x=x_{\text{0}}{\;C}^{T}$, with T = −1.701. The x-axis shows sleep cycle, and error bars indicate standard error. In panels B and C, each dot represents the SO percentage at a single time point (a 30-second epoch in B; a normalized time point in **C)**, and the brown line shows the smoothed SO percentage obtained by a running median across 10 consecutive time points.


We were firstly interested in estimating and modeling the cumulative trend of SO count decay across subsequent sleep cycles. For this analysis, we focused on the first four sleep cycles for each participant, since most participants had 4 or fewer cycles (Fig 1A) and cycles after the fourth had a very small amount of N3, which is the target of this modeling investigation. Consistent with known trends of SWA, we observed a decrease in the SO percentage during successive sleep cycles, both in each participant (Fig 1B shows one example) and in a normalized averaged representation of cycle-by-cycle SO emergence across all participants (Fig 1C). When considering the cumulative amount of SOs found within each cycle, Fig 1D shows again the decreasing trend across them, which we modeled with a power law relationship:

$$\begin{array}{c} x=x_{\text{0}}{\;C}^{T}\; \end{array}$$ (10)

where x represents the SO percentage in a given cycle (a cumulative count across all cycle divided by the total count of SOs in the night), $x_{\text{0}}$ is the SO percentage in the first cycle, C denotes the cycle number (1≤C≤4), and T, the rate of decay, is equal to -1.701. The RMSE of the model equaled 0.0085. We modeled the across-cycle trend for each participant with a power law separately and found that the average decay rate across participants was -1.664±1.002. Thus, the SO percentage in cycles 2–4 declined systematically relative to the first cycle, allowing cross-cycle dynamics to be described based on early-night measurements. To assess whether an exponential decay formulation—commonly used in sleep homeostasis models—provides a better description of the across-cycle SO decline, we compared the power-law model with an anchored exponential model using a single fitted parameter (S8 Fig). At the group level, the exponential model provided a slightly better fit (R² = 0.992 vs. 0.988; RMSE = 1.92 vs. 2.29), with a fitted decay rate of R = −1.151 (Equation 4). However, at the individual subject level, both models performed similarly (power-law: R² = 0.758 ± 0.370, RMSE = 7.74 ± 5.41; exponential: R² = 0.762 ± 0.364, RMSE = 7.97 ± 5.25), with the power-law yielding slightly lower subject-level RMSE and no significant differences in R² and RMSE between models (p > 0.6). Given this comparable performance, we retained the power-law formulation for its interpretability and its linearization in log–log space, which permits direct visual inspection of the decay exponent. Repeating this analysis for the combined N2&N3 stage revealed a qualitatively similar decreasing trend in SO percentage across cycles (S9 Fig), with a group-level decay rate of −1.14 (RMSE = 0.0064) and a mean per-subject decay rate of −1.21 ± 0.73. A paired comparison of individual decay rates between N3 and N2&N3 showed that the N3 decay was significantly steeper (t(21) = −3.96, p = 0.0008, 95% CI [−1.08, −0.34]; S10 Fig), consistent with the expectation that including N2 epochs — which carry fewer SOs — attenuates the apparent rate of decline. The inter-subject variance in decay rates was substantially smaller for N2&N3 (variance = 0.28) than for N3 (variance = 1.12), indicating greater cross-subject consistency in the combined-stage analysis.

To determine whether the observed power-law decay in SO percentage is driven by changes in sleep architecture, we examined the relationship between NREM stage duration and SO expression. N3 duration decreased markedly across cycles, while N2 duration increased (S11 Fig). Cross-sectional analyses (across participants within each cycle) revealed that N3 duration was strongly and consistently associated with SO percentage across all cycles (r = 0.81–0.91, all p < 0.0001; S12 Fig), whereas N2 duration showed weaker and less consistent relationships. Within-subject analyses further demonstrated that declines in N3 duration closely tracked reductions in SO percentage across cycles (mean r = 0.97, p < 0.0001; S13 Fig). To directly test whether N3 duration accounts for the across-cycle decay, we performed a mixed-effects mediation analysis. N3 duration accounted for 64.5% of the cycle-related decline in SO percentage, while a significant direct effect of cycle remained (β = -0.279, p < 0.0001), indicating partial mediation. Given that N3 is characterized by a higher prevalence of SOs, this result reflects a strong statistical association between N3 duration and SO expression.

### 3.2. Characterizing the temporal structure of SO occurrence

**Fig 2:**
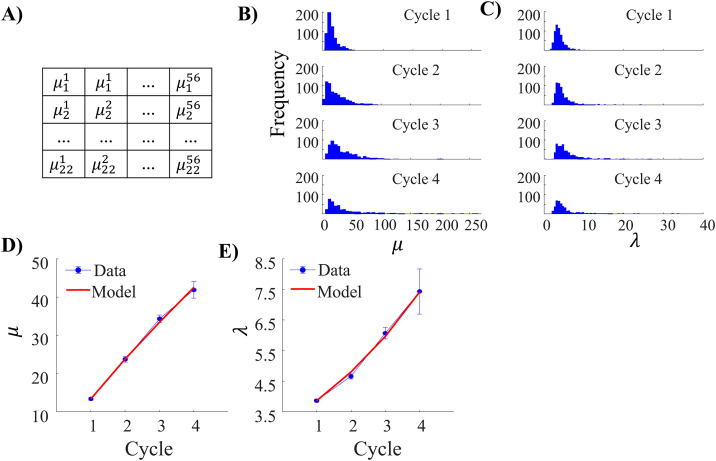
Inverse Gaussian (IG) model parameters for SO inter-arrival times across sleep cycles. A) Matrix of μ values across subjects (22) and EEG channels (56) for a single sleep cycle (totaling 1,232 values per parameter per cycle). B) Histogram of μ values across four sleep cycles. The x-axis shows μ, and the y-axis indicates frequency. A magnified inset is provided for the first cycle. C) Histogram of λ values across four cycles; inset shows the first cycle. D) Average μ across subjects and channels for each cycle. The trend follows a power law. E) Average λ across subjects and channels per cycle, modeled with an exponential fit. For panels D and E, averages were computed over 1,232 values per cycle (56 channels × 22 subjects). Error bars represent the standard error of the mean (SEM). Note that the SEM is substantially smaller than the full spread of values visible in panels B and C, which display the complete distribution of individual channel×subject parameter estimates. This distribution is right-skewed, particularly in later cycles where SO counts are lower, which causes the arithmetic mean in panels D and E to exceed the modal peak of the histograms in panels B and C.


To characterize the temporal structure of SO occurrence, we modeled the inter-arrival times of successive SOs and computed their cumulative sums to approximate SO timing within each cycle. Using the procedure outlined in Section 2.5, we obtained IG parameters (μ and λ) for each of the 56 EEG channels across 22 participants, resulting in a total of 56×22=1,232 parameter pairs per cycle. Fig 2A schematically shows the matrix of μ values for one sleep cycle. Separate matrices were generated for both μ and λ across all four cycles. Fig 2B and 2C illustrate the distribution of μ and λ values across cycles, respectively. The distributions of both parameters are right-skewed, particularly in later cycles where a subset of channel×subject combinations with low SO counts yielded high μ and λ estimates; consequently, the arithmetic mean reported in panels D and E exceeds the modal peak visible in the histograms of panels B and C. An increasing trend in the mean μ values across successive cycles was observed in Fig 2D, which we modeled using a power law.

$$\begin{array}{c} \mu =\mu_{\text{1}}\;C^{T}\; \end{array}$$ (11)

where $\mu_{\text{1}}$ represents the value of μ in the first cycle, C is cycle number, and T, which represents the increasing rate, was estimated as 0.8348. The RMSE of the model was 0.2381. By modeling μ for each participant separately, the average value of μ across participants was 0.831, with a variance of 0.44. Fig 2E shows the trends that emerged when the same procedure was applied to the distributions of λ values. The increasing trend in average λ across cycles was fitted using an exponential relationship as:


$$\begin{array}{c} \lambda =\lambda_{\text{1}}\;e^{\frac{C-\text{1}}{R}}\; \end{array}$$ (12)

where $\lambda_{\text{1}}$ represents the value of λ in the first cycle, C represents cycle number, and R represents the increasing rate estimated as 4.5893. The RMSE of the model was 0.0096. To justify the choice of functional form for both parameters, S14 Fig presents the cross-cycle trajectories of μ and λ on both linear and transformed scales, with power-law and exponential fits overlaid. For μ, the power-law fit appears as a straight line in log-log space while the exponential deviates, confirming the power-law as the better-fitting form. For λ, the exponential fit is linearized in log-linear space while the power-law deviates, supporting the exponential choice. This visual comparison directly motivates the functional form selected for each parameter. The parameter μ represents the mean inter-arrival time between successive SOs at a given electrode (in seconds). Its power-law increase across cycles means that SOs arrive progressively less frequently as the night advances — consistent with the well-established decline in slow-wave activity under diminishing homeostatic sleep pressure. The parameter λ governs the shape of the inverse Gaussian distribution: a larger λ corresponds to a narrower distribution of inter-arrival times around their mean, indicating more regular (less variable) SO timing. The exponential increase in λ across cycles therefore reflects a progressive regularization of SO occurrence: although fewer SOs occur in later cycles, those that do occur are more evenly spaced in time. Together, the increases in μ and λ capture a shift across the night from a high-density, highly variable SO regime — characteristic of maximal homeostatic drive in early sleep — toward a lower-density but more rhythmically predictable pattern in later cycles. By modeling λ individually for each participant, the mean value of λ across all participants was 8.506, with a variance of 0.113. Relationships 9 and 10 describe how the μ and λ parameters evolve across cycles in a systematic manner based on values observed during the first sleep cycle.

To assess whether the within-cycle temporal structure of SOs is preserved when N2 is included, we estimated the IG parameters μ and λ for the N2&N3 condition using the same renewal process procedure (S5 Table). Both parameters showed a monotonically increasing trend across cycles, consistent with N3 results. N2&N3 values were consistently larger: μ increased from 14.46 ± 0.41 s (Cycle 1) to 59.20 ± 2.25 s (Cycle 4), and λ from 4.10 ± 0.05 (Cycle 1) to 16.04 ± 1.90 (Cycle 4). Cycle-by-cycle paired comparisons revealed significant differences in μ in cycles 1 and 3, and in λ in cycles 1–3, indicating that while the absolute magnitude of SO timing structure is altered by the inclusion of N2, the cross-cycle trajectory is preserved. The larger μ in N2&N3 reflects lower SO density in N2 epochs; the larger λ indicates a more regular inter-arrival time distribution in the combined condition, likely reflecting the more homogeneous and lower-density SO distribution in N2 epochs, which reduces variability in inter-arrival times when combined with N3. To assess whether the observed model parameters reflect spatially meaningful differences in SO dynamics, we performed a regional analysis comparing frontal and posterior electrode groups. Frontal electrodes (FP1, FP2, FPz, F3, F4, Fz, F7, F8) exhibited consistently lower μ values compared to posterior electrodes (P3, P4, P1, P2, P5, P6, Pz, O1, O2, P7, PO3, PO4, POz, P8), indicating shorter inter-SO intervals and higher SO density at frontal sites (S15 Fig). Additionally, λ values were higher in frontal regions during early sleep cycles, suggesting more regular SO timing at frontal electrodes early in the night, with this difference diminishing across later cycles. Despite these regional differences in magnitude, both frontal and posterior groups showed similar across-cycle trajectories, with increasing μ and evolving λ across successive cycles. These results are consistent with the well-established frontal predominance of SOs and indicate that the model parameters capture regionally differentiated dynamics rather than purely global scalp averages.

To quantify model performance, we used three evaluation metrics: absolute difference error, distribution similarity (based on the KS test), and KL divergence (Table 1). To assess robustness, each metric was computed over 100 bootstrapped simulations. The analysis was conducted for cycles two through four using model parameters derived from the first cycle. Both absolute difference error and KL divergence increased across cycles, indicating a decline in model-data correspondence as the night progressed. To clarify the interpretation of these results, we emphasize that the model is not intended to provide precise pointwise prediction of SO timing in later cycles, but rather to capture partial temporal continuity in the statistical structure of SO occurrence across the night. In this context, distribution similarity was defined as the fraction of bootstrap simulations in which the KS test failed to reject the null hypothesis, indicating statistical indistinguishability between simulated and observed inter-arrival time distributions. Under the null hypothesis (α = 0.05), a baseline rejection rate of 0.05 is expected by chance. Therefore, distribution similarity values substantially greater than 0.05 indicate that the first-cycle model captures non-random structure in later-cycle SO timing. The observed values (Cycles 2–4: 0.51, 0.70, 0.63) reflect moderate but imperfect distributional correspondence between model-generated and empirical inter-arrival times. These values should be interpreted with the following caveat: because SO counts decline systematically across the night, later cycles provide fewer inter-arrival intervals per channel-subject combination, which reduces the statistical power of the KS test to detect distributional differences. This sample-size dependence means that the absolute values of distribution similarity are not directly comparable across cycles. Nevertheless, all three values substantially exceed the 0.05 chance baseline, confirming that the first-cycle model captures genuine non-random temporal structure in later cycles. The non-monotonic pattern — with similarity peaking in Cycle 3 before declining in Cycle 4 — likely reflects the combined influence of declining SO counts (which reduces KS test sensitivity and mechanically inflates apparent similarity in Cycle 3) and the genuine increase in inter-individual variability by Cycle 4, which degrades distributional correspondence beyond what the sample-size effect alone would predict. Notably, distribution similarity peaked during the third cycle, suggesting relatively stronger alignment between simulated and observed SO timing in that interval. Although distribution similarity was highest in the third cycle, visual comparison of the parameter trends in Fig 2D–2E may suggest a larger apparent discrepancy between the model and empirical data relative to cycle 2. This occurs because the similarity metric in Table 1 evaluates the correspondence between the distributions of simulated and empirical inter-arrival times, whereas Fig 2D–2E illustrate cycle-averaged parameter trends (μ and λ). Consequently, small deviations in the averaged parameter trajectories may appear visually larger even when the underlying inter-arrival time distributions remain closely aligned.

**Table 1:** Absolute difference error, distribution similarity and Kullback-Leibler divergence using 100 times bootstrapping.

	Cycle 2	Cycle 3	Cycle 4
Absolute difference Error	0.1774±0.0029	0.1939±0.0030	0.2941±0.0067
Distribution similarity (%)	0.5079±0.0398	0.7061±0.0435	0.6343±0.0346
Kullback-Leibler divergence	0.0092±0.0008	0.0118±0.0013	0.0124±0.0011

**Fig 3:**
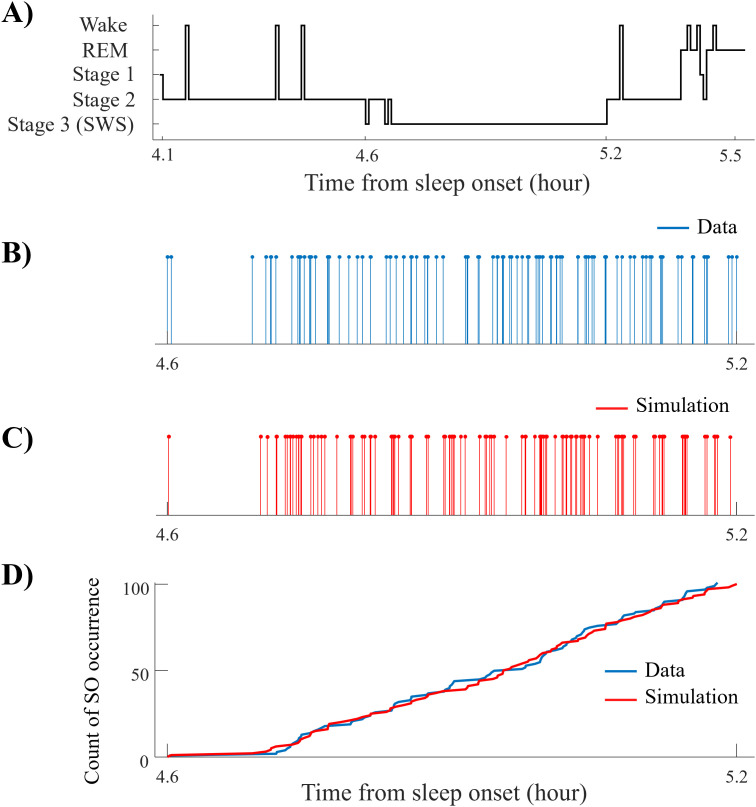
Simulated and observed SO timing in EEG channel Fz during the third sleep cycle of one participant. A) Hypnogram from sleep onset through the end of the third cycle. The x-axis represents time (in hours); wake periods correspond to EEG epochs marked by major body movements. B) Detected SOs in the original EEG recording for this cycle (n = 101). C) Simulated SO occurrences generated using μ = 8.93 and λ = 3.87. D) Comparison of SO timing between the empirical data and one simulation run for the third cycle.

To visually demonstrate model performance, Fig 3 presents a simulation of SO timing during the third cycle of Participant 4, for a single EEG channel. This example illustrates modeled SO occurrences based on inter-arrival times generated from the fitted distribution. The μ and λ parameters used in the simulation were estimated from the participant’s first cycle and extrapolated to the third cycle using Equations 9 and 10. Simulated SO intervals were generated by transforming uniform random values via the inverse CDF, following the procedure described in Section 2.5. This example reflects the stochastic nature of SO timing and provides a representative case of how the model reproduces observed patterns in spontaneous sleep data.

**Fig 4:**
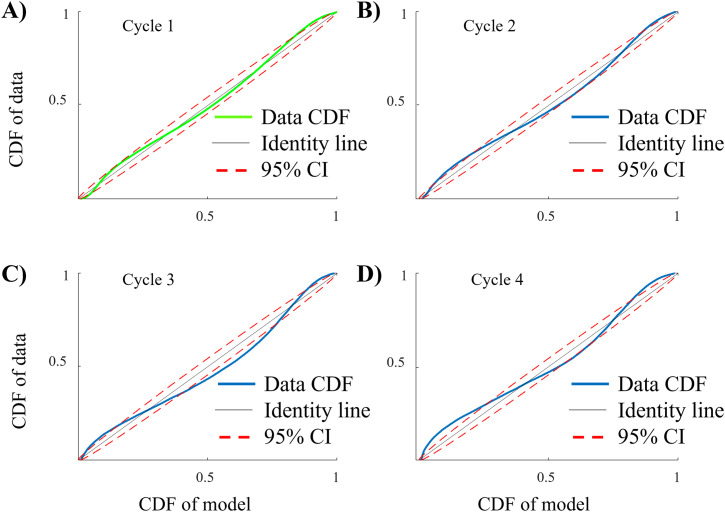
Probability–probability (P–P) plots comparing cumulative distributions of observed and simulated SO inter-arrival times across sleep cycles. The y-axis shows the empirical cumulative distribution function (CDF); the x-axis shows the model-generated CDF. The red dashed line represents the 95% confidence interval. Panels A–D display results for cycles 1 through 4, respectively. The black diagonal line represents the identity line (y = x), indicating perfect agreement between model and data. In Panel A (green), the IG model was directly fit to the inter-arrival times from the first cycle. In Panels B–D (blue), the model parameters were extrapolated from first-cycle estimates using Equations 9 and 10.

We also used probability–probability (P–P) plots to evaluate the alignment between the model-generated and empirical distributions of SO inter-arrival times across successive sleep cycles. Following the procedure outlined in Section 2.6, within each cycle, the length of the CDF of both the data and the model was equalized to 250 points and then averaged across all participants. In the resulting P–P plots, closer alignment to the diagonal line indicates greater similarity between the empirical and modeled distributions. As shown in Fig 4, the majority of points fell within the 95% confidence interval, supporting an acceptable goodness of fit between the model and observed data.

## 4. Discussion

This study presents a new approach to characterizing when SOs occur during sleep, using information from the first sleep cycle to model their timing across the night. We introduce a modeling approach that characterizes SO emergence across the sleep night based on parameters derived from activity in the first sleep cycle. By modeling how SOs evolve over successive cycles and scalp regions, we can identify patterns that are consistent across individuals and those that vary by person or night. This perspective reframes SOs not as isolated events, but as expressions of a structured stochastic process with both population-level regularities and individual variability.

Our model captures how SOs gradually decrease in density (events per second) across sleep cycles as the night progresses, using a power law function dependent on the SO percentage in the first cycle. We model the specific timing of SOs within each cycle using point processes built on the distribution of SO inter-arrival times (represented with IG). Data showed that parameters μ and λ in the IG model changed across sleep cycles with an increasing trend, which we modeled with power law and exponential relationships, respectively. This study is fundamentally focused on identifying trends in SO timing through probabilistic modeling, offering a theoretical framework for understanding the temporal dynamics of SO occurrences. The evaluation methods employed, including goodness-of-fit analyses and cumulative distribution functions, are tailored to assess how well the model reflects observed trends. By centering on these modeling objectives, this study provides a foundation for future research and underscores the potential of probabilistic approaches to enhance our understanding of SO dynamics, establishing an approach that can be extended to other sleep events (e.g., spindles).

Previous research has shown an exponential decreasing trend in the power of SWA across successive cycles [55], which serves as an index of sleep pressure (Process S) in sleep homeostasis and is well modeled in the literature [56–60]. Since fundamental dynamics of SOs contribute to the power of SWA, we expected to observe a reduced count of SOs in successive cycles. This study expands on the SWA-based knowledge to confirm substantially similar trends in SOs and to explicitly quantify the relationships between SO counts across subsequent sleep cycles. Both IG parameters can be related to the two-process model of sleep regulation. The power-law increase in μ (mean inter-SO interval) parallels the exponential decline in SWA power across NREM periods that serves as the empirical index of homeostatic sleep pressure (Process S). As homeostatic drive diminishes, SOs become progressively less frequent — captured here as increasing μ — providing an event-level correlate of the SWA-based homeostatic index. The power-law exponent of μ is consistent with the decelerating rate of SWA dissipation observed in later cycles. The exponential increase in λ captures a distinct dimension of overnight dynamics not represented in standard two-process formulations: the progressive regularization of SO timing as homeostatic drive wanes. High λ in later cycles reflects a reduction in inter-SO variability despite lower SO density, suggesting a shift from a high-drive, variable-burst regime in early sleep to a lower-drive, more rhythmically predictable regime in later cycles — consistent with the known reduction in SO clustering in later NREM periods and with ultradian models of sleep architecture. In addition to the overall decline in SO percentage across successive sleep cycles, Fig 1C reveals a non-monotonic profile within the first cycle, characterized by an initial increase in SO proportion, a transient reduction, and a subsequent rise before the first REM transition. One possible explanation is that this pattern reflects the interaction between high homeostatic sleep pressure at sleep onset and the evolving internal structure of the first NREM period. Early-night sleep is known to contain the greatest concentration of slow-wave activity, and the first NREM period is particularly prominent in this regard [61]. Consistent with this framework, the initial peak may reflect rapid entry into deeper NREM sleep under maximal homeostatic drive, whereas the intermediate dip and later rebound may arise from fluctuations in sleep depth as the brain transitions between lighter and deeper NREM stages prior to REM onset [62]. This interpretation is consistent with the known internal architecture of NREM sleep periods, in which N3—characterized by a higher density of slow oscillations compared with N2—does not occur as a monolithic block but is interspersed with lighter N2 stages [63]. Furthermore, with sleep progression the composition of NREM sleep changes systematically across cycles, with deep slow-wave sleep concentrated earlier in the night and lighter NREM stages becoming more prevalent later [37]. Although the present study was not designed to directly test the mechanistic basis of this within-cycle pattern, the observation suggests that SO production within the first cycle is not temporally uniform. Instead, it may reflect the heterogeneous composition of ‘slow wave tides’—the internal waxing and waning of SWA subtypes driven by shifting sleep depth between stages N2 and N3 during early-night NREM sleep ^63^.

The analyses of NREM stage dynamics clarify that the power-law decay of SOs is strongly associated with the progressive reduction of N3 sleep across cycles (S11–S13 Figs). However, this relationship should be interpreted in light of the fact that sleep staging provides a coarse categorization of underlying electrophysiological activity, and N3 is intrinsically defined by a higher density of slow oscillations. As such, the strong association between N3 duration and SO percentage likely reflects their shared physiological basis rather than a unidirectional causal mechanism. The persistence of a significant direct effect of sleep cycle indicates that SO dynamics are not fully explained by stage composition alone. Instead, the probability of SO occurrence within NREM sleep appears to decrease across the night independently of total N3 duration, suggesting the involvement of additional homeostatic or network-level processes not captured by conventional staging. These findings support the view that SO emergence is governed by continuous temporal dynamics, of which sleep stage labels provide only a partial and discretized representation.

Our findings have implications for research in brain stimulation during sleep that is timed to SO online detection. By modeling SO inter-arrival dynamics and characterizing structured variability across cycles and individuals, our framework offers a complementary perspective to current real-time detection approaches. Specifically, μ — the mean inter-SO interval — could serve as a cycle-specific prior for setting the expected inter-stimulation interval in closed-loop systems, while λ — which governs the regularity of SO timing — could inform the width of the detection window used to anticipate the next SO. Together, these parameters may help reduce missed stimulation opportunities in later cycles, where SO density is lower and timing is more regular. The probabilistic structure uncovered here could therefore inform the design of cycle-adaptive stimulation protocols that adjust trigger timing based on early-night parameter estimates rather than fixed thresholds.

Translational contributions of the proposed modeling approach abound, given it can be applied to individual nights, and therefore both to longitudinal studies of sleep and studies in clinical populations. Of note, since the modeling is applied to each electrode, data-driven estimation of fit parameters can be applied to sleep acquisitions in low density EEG, which are common in clinical sleep laboratories. Given the pivotal role of SOs in cognition [17], deviations from typical SO timing trends — such as those observed across sleep cycles — may provide insight into cognitive disorders or neurodevelopmental conditions. More broadly, this descriptive model of SO emergence supports a deeper understanding of individualized temporal structure that could guide future designs in both experimental and clinical contexts.

From a practical standpoint, the present results inform recommendations for future probabilistic modeling of SO temporal dynamics. In healthy adults with full-night recordings, N3 is recommended as the primary staging for this type of analysis: the steeper power-law exponent and more physiologically interpretable parameter trajectories make N3-based models more sensitive to the homeostatic processes that drive SO generation. However, the N2&N3 condition demonstrated substantially smaller inter-subject variance in decay rates (SD = 0.53 vs. 1.06 in N3), producing more consistent estimates across individuals. This reduced variability may be advantageous when N3 duration is limited — as is common in older adults, clinical populations with disrupted sleep architecture, or short nap paradigms — where N3-only modeling may be underpowered. In such contexts, N2&N3 provides a viable and statistically stable alternative, at the cost of a shallower and less stage-specific decay model. Researchers should therefore consider the trade-off between physiological specificity (favoring N3) and cross-participant robustness (favoring N2&N3) when selecting a staging approach.

The current study has several limitations. The dataset comprised 22 participants (9 females), limiting our ability to examine demographic effects such as age or sex without compromising statistical power. We also restricted our analysis to the first four sleep cycles due to sparse N3 content later in the night, which may limit applicability to late-night sleep. The model currently averages parameters across all EEG channels with equal weight, without accounting for the known frontal dominance of SOs [64]. Weighted spatial modeling that prioritizes frontal electrodes may improve the biological interpretability of the model. Future work could examine whether the structured temporal dynamics identified here differ systematically across scalp regions, which would complement the spatially weighted modeling approach described above. Additionally, the dataset is restricted to young healthy adults (18–34 years), which limits generalizability to older adults or individuals with sleep disorders such as Hypersomnolence, in whom SO dynamics differ substantially [11]. While a sample size of 22 individuals is consistent with prior EEG sleep studies characterizing SO dynamics [29,30,65], future work should validate this framework in larger and more demographically diverse cohorts to assess the robustness of model parameters across populations and to characterize inter-individual variability as a function of relevant characteristics (e.g., age, sex, hormonal levels). Furthermore, the renewal process framework applied here assumes that SO inter-arrival times are independent draws from a stationary distribution within each NREM cycle — that is, the SO generation rate does not vary systematically over time within a cycle. While this approximation is reasonable for early cycles dominated by stable N3 sleep, it becomes increasingly tenuous in later cycles, where declining homeostatic drive and a greater proportion of lighter N2 sleep may introduce within-cycle non-stationarity. The progressive increase in the SEM of both μ and λ from Cycle 1 to Cycle 4 (S5 Table) is consistent with this interpretation, reflecting growing parameter uncertainty as the stationarity assumption is less well supported. The assumption of conditional independence between successive SO intervals is likewise an idealization, and future work incorporating non-stationary or self-exciting point process models (e.g., inhomogeneous Poisson or Hawkes processes) could provide a more flexible framework for capturing such dependencies and better reflect individual EEG dynamics [66,67]. Additionally, the minimum inter-event interval used as a lower bound in the simulation was defined channel-wise from the observed data but averaged across subjects for application in the model; this averaging may obscure meaningful spatial and individual differences in SO trough temporal spacing that future work should characterize explicitly. Overall, this model provides a framework for characterizing the temporal dynamics of SOs across the sleep night, and with further validation, it can support individualized modeling approaches for future translational research.

## Supporting information

S1 Table Sleep outcomes for our 22 participants. WASO: wake after sleep onset. For each value, time is reported in minutes.
(DOCX)

S2 Table Summary statistics of SO properties across sleep stages. Reported values include total event count, mean ± standard deviation, and median for duration, trough amplitude, down-state slope, and up-state slope across N2, N3, and combined NREM (N2 + N3).
(DOCX)

S3 Table Summary statistics of SO properties across sleep cycles (Cycles 1–4) during N3 sleep. Reported values include total event count, mean, and median for duration, trough amplitude, down-state slope, and up-state slope.
(DOCX)

S4 Table Performance of seven functions in modeling the inter-arrival times of SO occurrences in our dataset. We evaluated the performance of functions by calculating the ratio of p-values from the KS-test greater than 0.05 over 100 data simulations. For each data simulation within each cycle, we fitted the function to the data of that cycle. Values closer to 1 indicate a higher similarity between the data and the model. As can be seen, the inverse Gaussian function shows the highest value among the other functions.
(DOCX)

S5 Table IG model parameters (μ and λ) for N3 and N2&N3 conditions across sleep cycles. Values are pooled mean ± SEM across all channels and subjects. Paired t-tests compare N3 vs. N2&N3 at the subject level (averages over channels). * p < 0.05.
(DOCX)

S1 Fig Distributions of SO properties across NREM sleep (N2 + N3). (A) Duration, (B) trough amplitude, (C) down-state slope, and (D) up-state slope. Red and black vertical lines indicate median and mean values, respectively.
(TIF)

S2 Fig Comparison of SO properties between sleep stages N2 and N3. Boxplots show distributions of (A) duration, (B) trough amplitude, (C) down-state slope, and (D) up-state slope. Colored markers indicate mean values.
(TIF)

S3 Fig SO properties across sleep cycles (Cycles 1–4) during N3 sleep. Boxplots show distributions of (A) duration, (B) trough amplitude, (C) down-state slope, and (D) up-state slope across cycles. Colored markers indicate mean values.
(TIF)

S4 Fig Anterior-to-posterior gradient of SO properties during combined N2 and N3 sleep. SO properties—including duration, trough amplitude, down-state slope, and up-state slope—are shown across four electrode groups (Frontal, Central, Parietal, Occipital). Values represent the mean of per-subject medians ± SEM. A clear anterior-to-posterior gradient is observed, with frontal regions exhibiting shorter durations and larger amplitudes compared to posterior regions.
(TIF)

S5 Fig Anterior-to-posterior gradient of SO properties across sleep cycles in N3 sleep. SO properties (duration, trough amplitude, down-state slope, and up-state slope) are shown across four electrode groups (Frontal, Central, Parietal, Occipital), separately for each sleep cycle during N3. Values represent the mean of per-subject medians ± SEM. The anterior-to-posterior gradient is preserved across cycles.
(TIF)

S6 Fig Comparison of anterior-to-posterior gradients of SO properties between N2 and N3 sleep. SO properties (duration, trough amplitude, down-state slope, and up-state slope) are compared between N2 and N3 sleep stages across four electrode groups (Frontal, Central, Parietal, Occipital). Values represent the mean of per-subject medians ± SEM.
(TIF)

S7 Fig Average count of 30s EEG epochs across participants in sleep cycles. x-axis shows first four sleep cycles number and error bar shows standard error.
(TIF)

S8 Fig Comparison of power-law and exponential models for SO percentage across sleep cycles. A) Group-level N3 SO percentage across four sleep cycles with fitted power-law (blue) and exponential (red dashed) models shown on a linear scale. The exponential model yields a marginally better group-level fit (R² = 0.992 vs. 0.988; RMSE = 1.92 vs. 2.29). B) Log–log representation of the same data, demonstrating the linearization of the power-law model and enabling direct visual assessment of the decay exponent. Both models are anchored to the observed Cycle 1 mean (x₀ = 59.6%) and have a single free parameter. Error bars indicate ±SEM across subjects.
(TIF)

S9 Fig Total SO percentage during N2&N3 across successive cycles by averaging SO percentage across all participants. x-axis shows sleep cycle and error bar shows standard error. The decreasing trend in SO percentage across cycles can be described by a power law model with decreasing rate equal to -1.139. The RMSE of the model is 0.0064.
(TIF)

S10 Fig Per-subject power-law decay exponents for SO percentage across sleep cycles: N3 vs. N2&N3. Each point represents one participant’s fitted power-law exponent (T) for either N3-only (blue circles) or combined N2&N3 (red squares). Gray lines connect paired observations within each participant. Black error bars show group mean ± SD. N3 decay was significantly steeper (paired t-test: t(21) = −3.956, p = 0.0008; n = 22 subjects).
(TIF)

S11 Fig Evolution of NREM stage durations across sleep cycles. Mean duration (± standard error) of N2, N3, and combined N2 + N3 sleep stages across the first four sleep cycles. N3 duration shows a progressive decline across cycles, while N2 duration increases, reflecting the well-established redistribution of NREM sleep architecture across the night. Total NREM (N2 + N3) duration exhibits a gradual decrease. Each point represents the average across participants, with error bars indicating standard error of the mean.
(TIF)

S12 Fig Cross-sectional relationship between NREM stage duration and SO percentage. Scatter plots showing the relationship between stage duration (N2, N3, and N2 + N3) and SO percentage across participants for each sleep cycle. Each point represents one participant. N3 duration exhibits a strong positive correlation with SO percentage across all cycles, whereas N2 duration shows weaker and less consistent relationships. Solid lines indicate linear regression fits.
(TIF)

S13 Fig Within-subject association between NREM stage duration and SO percentage across cycles. Distribution of within-subject Pearson correlation coefficients (r) quantifying the relationship between stage duration and SO percentage across sleep cycles. Each value represents one participant. N3 duration shows a consistently strong positive relationship with SO percentage across individuals, indicating that reductions in N3 duration closely track decreases in SO expression within subjects.
(TIF)

S14 Fig Model selection for cross-cycle parameter trajectories. Left column: μ (top) and λ (bottom) plotted on linear scale with power-law (red solid) and exponential (blue dashed) fits overlaid. Right column: log-log scale for μ and log-linear scale for λ, where the preferred model (power-law for μ, exponential for λ) appears as a straight line. Error bars indicate SEM.
(TIF)

S15 Fig Regional comparison of inverse Gaussian parameters across sleep cycles. A) Mean μ (inter-SO interval, seconds) and (B) mean λ (shape parameter) across sleep cycles for frontal (FP1, FP2, FPz, F3, F4, Fz, F7, F8) and posterior (P3, P4, P1, P2, P5, P6, Pz, O1, O2, P7, PO3, PO4, POz, P8) electrode groups. Values are shown as mean ± SEM across subjects, with individual subject trajectories displayed in light red (posterior) and blue (frontal). Frontal electrodes exhibit consistently lower μ values, indicating higher SO density, and higher λ values in early cycles, suggesting more regular SO timing. Both regions show similar across-cycle trends. Asterisks indicate significant differences between regions (paired t-test, p < 0.05).
(TIF)
