# Supplementary material for: Sleep slow oscillation emergence on the scalp as a renewal point process
Source: PLoS Comput Biol. 2026 Jul 29;22(7):e1014572. doi: 10.1371/journal.pcbi.1014572 (PMC13432095; doi:10.1371/journal.pcbi.1014572)
Supplement: S1 Table — WASO: wake after sleep onset. For each value, time is reported in minutes. (DOCX) [file pcbi.1014572.s001.docx]

| Total Sleep Time | $465.36\pm7.76$ |
| --- | --- |
| N1 | $22.1591\pm10.40$ |
| N2 | $199.75\pm25.92$ |
| N3 | $126.68\pm31.61$ |
| REM | $102.07\pm14.96$ |
| Sleep Onset | $14.04\pm6.71$ |
| WASO | $5.30\pm4.75$ |
