# Supplementary material for: Sleep slow oscillation emergence on the scalp as a renewal point process
Source: PLoS Comput Biol. 2026 Jul 29;22(7):e1014572. doi: 10.1371/journal.pcbi.1014572 (PMC13432095; doi:10.1371/journal.pcbi.1014572)
Supplement: S2 Table — Reported values include total event count, mean ± standard deviation, and median for duration, trough amplitude, down-state slope, and up-state slope across N2, N3, and combined NREM (N2 + N3). (DOCX) [file pcbi.1014572.s002.docx]

| **Property** | **NREM** | **N2** | **N3** |
| --- | --- | --- | --- |
| Count | 1,172,471 | 251,395 | 921,076 |
| Median duration (s) | 1.07 | 1.21 | 1.05 |
| Mean duration (s) | $1.21\pm0.58$ | $1.45\pm0.81$ | $1.15\pm0.$48 |
| Median trough amplitude (μV) | -133.69 | -145.25 | -130.84 |
| Mean trough amplitude (μV) | $-148.68\pm63.03$ | $-163.93\pm69.59$ | $-144.52\pm60.45$ |
| Median Before-trough slope (μV/s) | 587.64 | 565.84 | 594.08 |
| Mean Before-trough slope (μV/s) | 685.89$\pm$445.69 | 668.38$\pm$499.37 | 690.68$\pm$429.75 |
| Median After-trough slope (μV/s) | 170.57 | 160.44 | 172.82 |
| Mean After-trough slope (μV/s) | 199.32$\pm$124.96 | 201.32$\pm$149.50 | 198.78$\pm$117.37 |
