# Supplementary material for: Sleep slow oscillation emergence on the scalp as a renewal point process
Source: PLoS Comput Biol. 2026 Jul 29;22(7):e1014572. doi: 10.1371/journal.pcbi.1014572 (PMC13432095; doi:10.1371/journal.pcbi.1014572)
Supplement: S3 Table — Reported values include total event count, mean, and median for duration, trough amplitude, down-state slope, and up-state slope. (DOCX) [file pcbi.1014572.s003.docx]

| **Property** | **C1 Mean** | **C1 Median** | **C2 Mean** | **C2 Median** | **C3 Mean** | **C3 Median** | **C4 Mean** | **C4 Median** |
| --- | --- | --- | --- | --- | --- | --- | --- | --- |
| Count | 551992 | | 201065 | | 93678 | | 53682 | |
| Duration (s) | 1.08 | 1.00 | 1.20 | 1.10 | 1.28 | 1.15 | 1.27 | 1.13 |
| Trough amplitude (µV) | -141.39 | -129.25 | -145.55 | -131.51 | -151.40 | -133.46 | -156.06 | -138.33 |
| Before-trough slope (µV/s) | 705.38 | 610.69 | 664.39 | 569.66 | 658.98 | 557.04 | 692.31 | 586.55 |
| After-trough slope (µV/s) | 203.78 | 178.08 | 188.93 | 164.45 | 189.45 | 160.57 | 197.34 | 169.51 |
