# Supplementary material for: Sleep slow oscillation emergence on the scalp as a renewal point process
Source: PLoS Comput Biol. 2026 Jul 29;22(7):e1014572. doi: 10.1371/journal.pcbi.1014572 (PMC13432095; doi:10.1371/journal.pcbi.1014572)
Supplement: S4 Table — We evaluated the performance of functions by calculating the ratio of p-values from the KS-test greater than 0.05 over 100 data simulations. For each data simulation within each cycle, we fitted the function to the data of that cycle. Values closer to 1 indicate a higher similarity between the data and the model. As can be seen, the inverse Gaussian function shows the highest value among the other functions. (DOCX) [file pcbi.1014572.s004.docx]

| Function | Cycle 1 | Cycle 2 | Cycle 3 | Cycle 4 | Average across cycles |
| --- | --- | --- | --- | --- | --- |
| Exponential | 0.02 | 0.19 | 0.79 | 0.600 | 0.386 |
| Gamma | 0.10 | 0.53 | 0.93 | 0.860 | 0.618 |
| Inverse Gaussian | 0.31 | 0.71 | 0.90 | 0.880 | **0.708** |
| Logistic | 0.10 | 0.58 | 0.86 | 0.830 | 0.598 |
| Normal | 0.18 | 0.70 | 0.93 | 0.910 | 0.694 |
| Poisson | 0.03 | 0.55 | 0.90 | 0.870 | 0.592 |
| Weibull | 0.06 | 0.63 | 0.92 | 0.900 | 0.640 |
