# Supplementary material for: Sleep slow oscillation emergence on the scalp as a renewal point process
Source: PLoS Comput Biol. 2026 Jul 29;22(7):e1014572. doi: 10.1371/journal.pcbi.1014572 (PMC13432095; doi:10.1371/journal.pcbi.1014572)
Supplement: S5 Table — Values are pooled mean ± SEM across all channels and subjects. Paired t-tests compare N3 vs. N2&N3 at the subject level (averages over channels). * p < 0.05. (DOCX) [file pcbi.1014572.s005.docx]

| **Cycle** | **μ** | | | | **λ** | |  |  |
| --- | --- | --- | --- | --- | --- | --- | --- | --- |
|  | N3 | **N2+N3** | **t-value** | **p-value** | N3 | **N2+N3** | **t-value** | **p-value** |
| C1 | 13.73 ± 0.40 | 14.46 ± 0.41 | −2.35 | **0.030 *** | 3.91±0.05 | 4.10 ± 0.05 | −2.86 | **0.010 *** |
| C2 | 24.63 ± 0.72 | 29.54 ± 0.88 | −0.40 | 0.695 | 4.79±0.09 | 6.05 ± 0.15 | −3.29 | **0.004 *** |
| C3 | 35.57 ± 0.99 | 46.58 ± 1.42 | −2.60 | **0.020 *** | 6.35±0.20 | 9.20 ± 0.49 | −3.43 | **0.004 *** |
| C4 | 42.53 ± 2.03 | 59.20 ± 2.25 | −0.38 | 0.714 | 7.28±0.68 | 16.04 ± 1.90 | −1.43 | 0.182 |
