# Supplementary figures and images for: Sleep slow oscillation emergence on the scalp as a renewal point process
Source: PLoS Comput Biol. 2026 Jul 29;22(7):e1014572. doi: 10.1371/journal.pcbi.1014572 (PMC13432095; doi:10.1371/journal.pcbi.1014572)

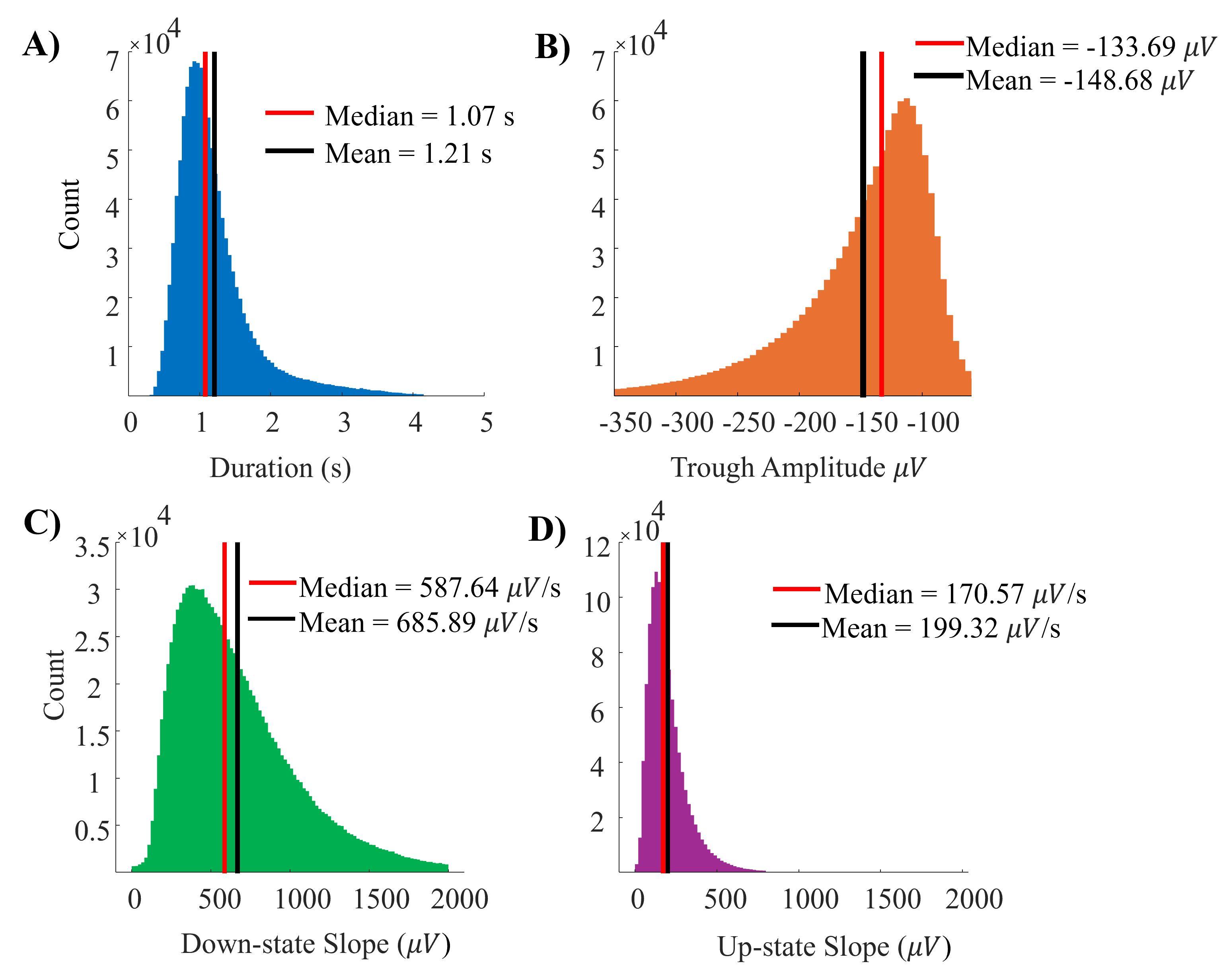

Supplement: S1 Fig — (A) Duration, (B) trough amplitude, (C) down-state slope, and (D) up-state slope. Red and black vertical lines indicate median and mean values, respectively. (TIF) [file pcbi.1014572.s006.tif]

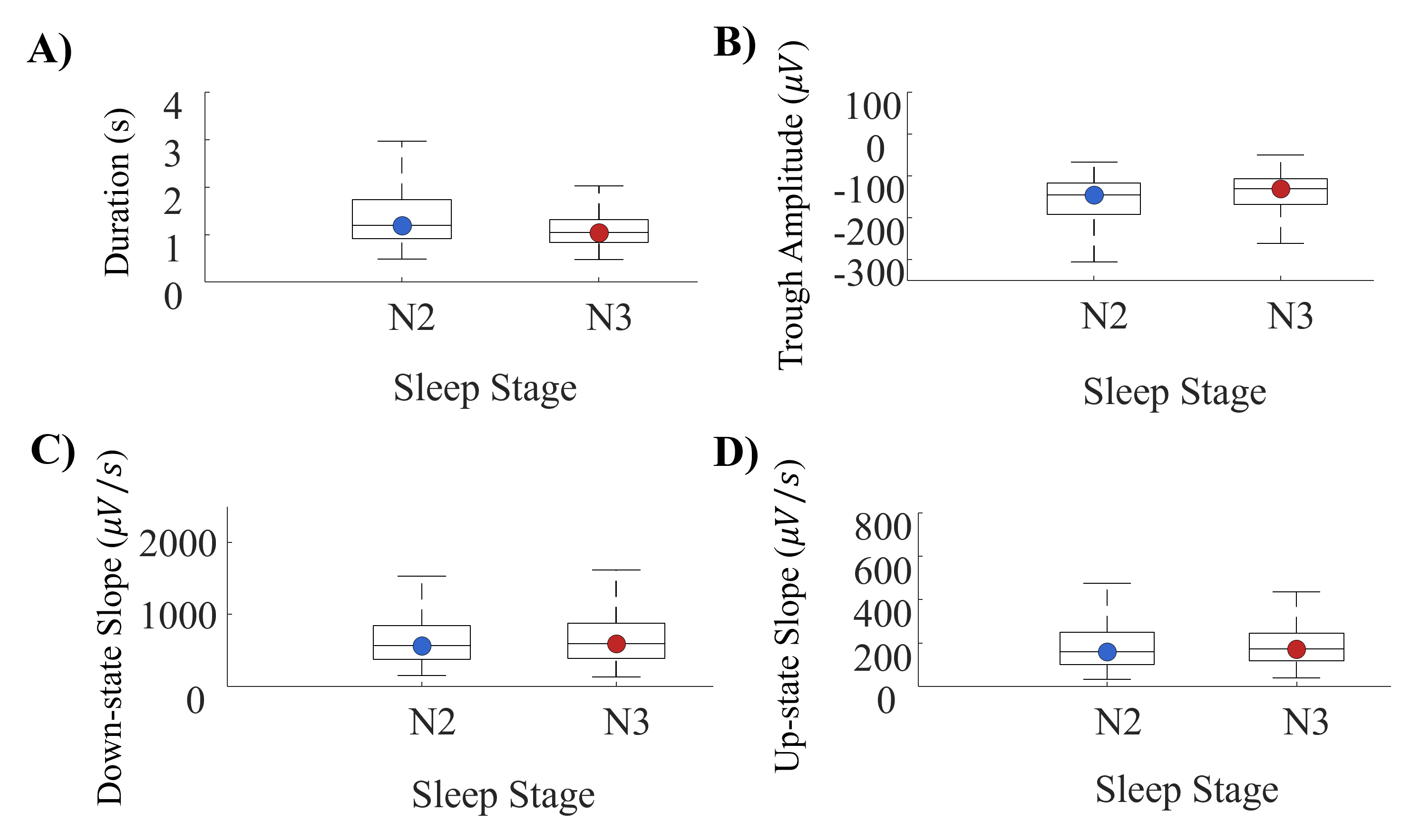

Supplement: S2 Fig — Boxplots show distributions of (A) duration, (B) trough amplitude, (C) down-state slope, and (D) up-state slope. Colored markers indicate mean values. (TIF) [file pcbi.1014572.s007.tif]

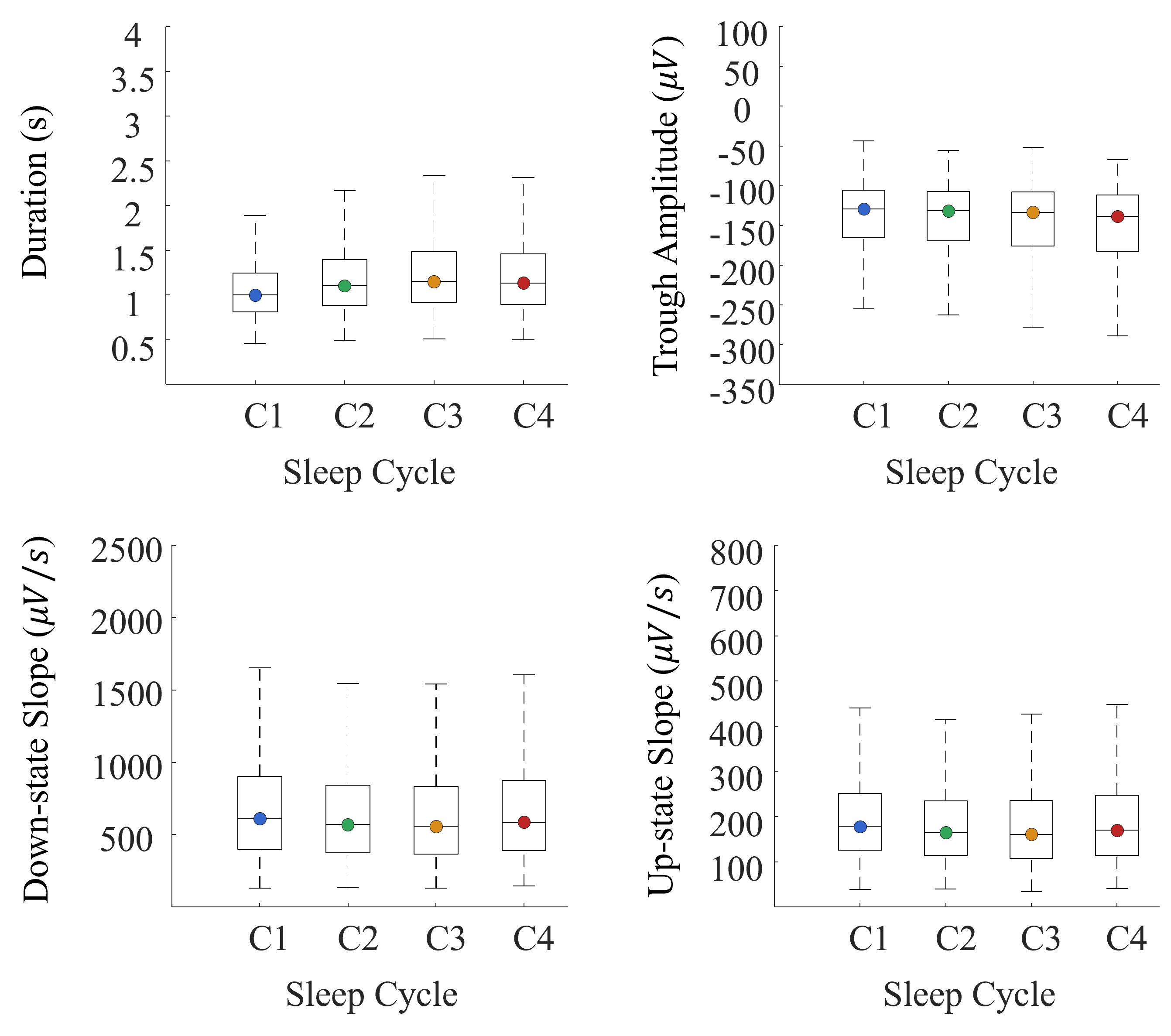

Supplement: S3 Fig — Boxplots show distributions of (A) duration, (B) trough amplitude, (C) down-state slope, and (D) up-state slope across cycles. Colored markers indicate mean values. (TIF) [file pcbi.1014572.s008.tif]

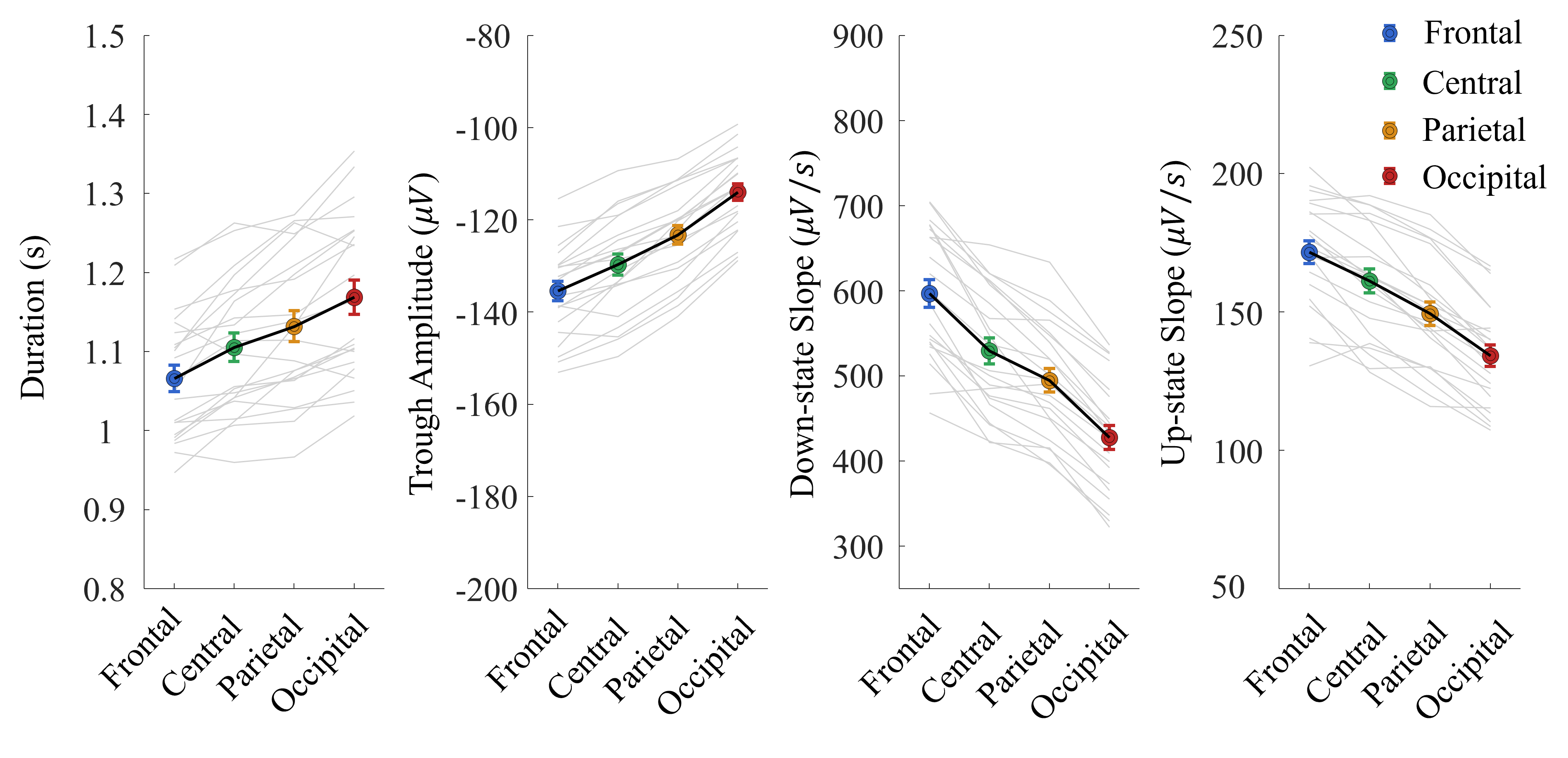

Supplement: S4 Fig — SO properties—including duration, trough amplitude, down-state slope, and up-state slope—are shown across four electrode groups (Frontal, Central, Parietal, Occipital). Values represent the mean of per-subject medians ± SEM. A clear anterior-to-posterior gradient is observed, with frontal regions exhibiting shorter durations and larger amplitudes compared to posterior regions. (TIF) [file pcbi.1014572.s009.tif]

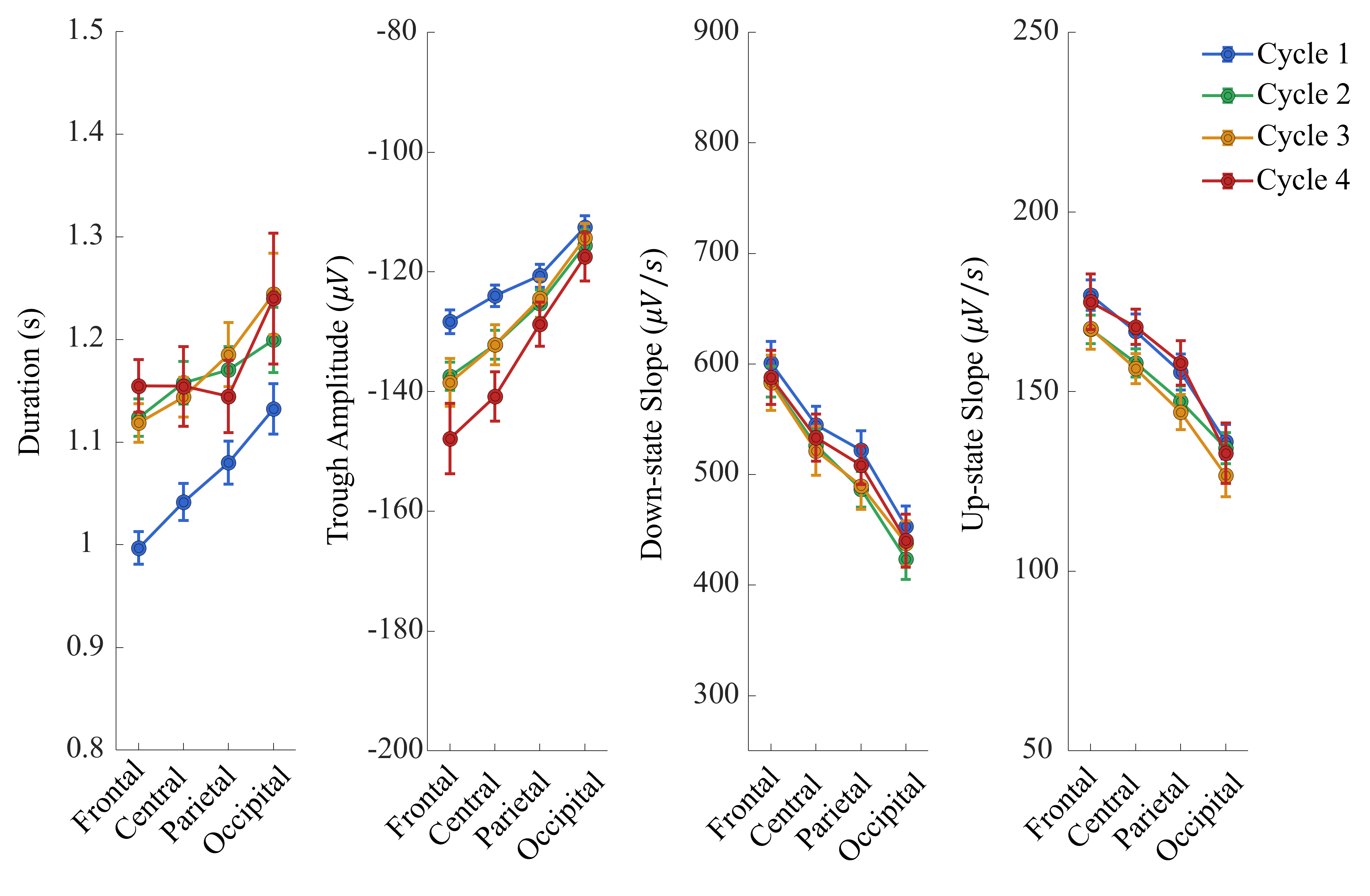

Supplement: S5 Fig — SO properties (duration, trough amplitude, down-state slope, and up-state slope) are shown across four electrode groups (Frontal, Central, Parietal, Occipital), separately for each sleep cycle during N3. Values represent the mean of per-subject medians ± SEM. The anterior-to-posterior gradient is preserved across cycles. (TIF) [file pcbi.1014572.s010.tif]

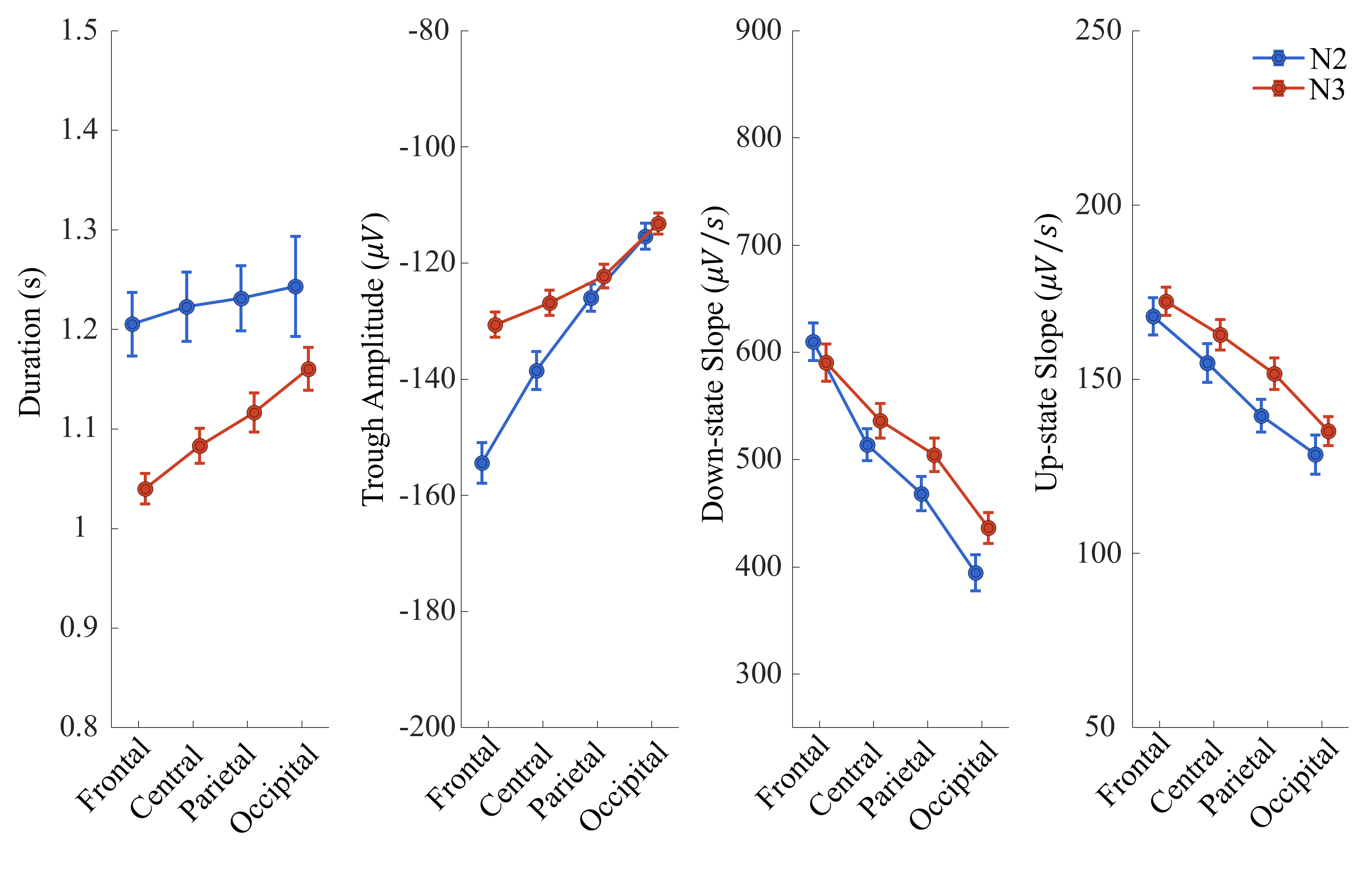

Supplement: S6 Fig — SO properties (duration, trough amplitude, down-state slope, and up-state slope) are compared between N2 and N3 sleep stages across four electrode groups (Frontal, Central, Parietal, Occipital). Values represent the mean of per-subject medians ± SEM. (TIF) [file pcbi.1014572.s011.tif]

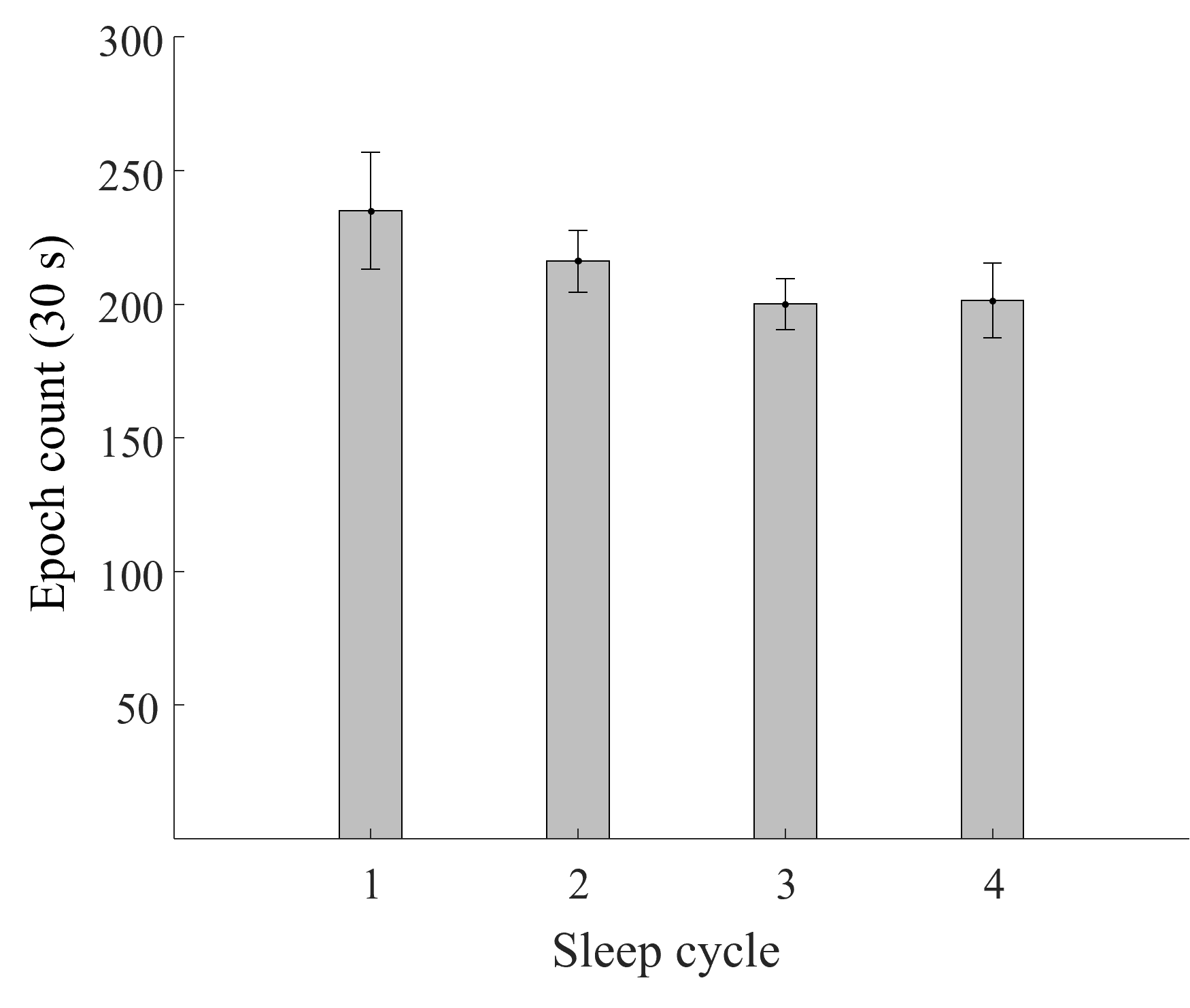

Supplement: S7 Fig — x-axis shows first four sleep cycles number and error bar shows standard error. (TIF) [file pcbi.1014572.s012.tif]

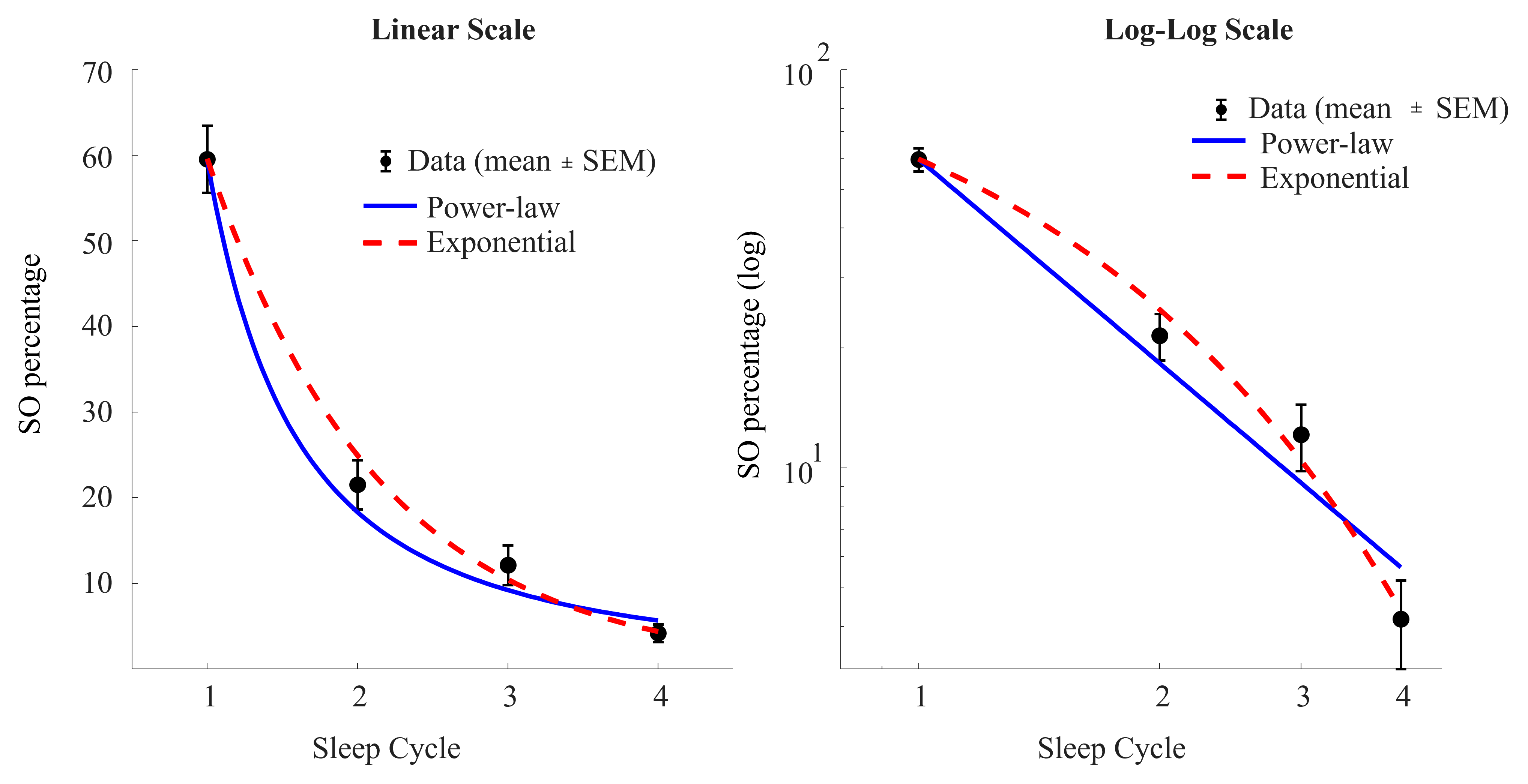

Supplement: S8 Fig — A) Group-level N3 SO percentage across four sleep cycles with fitted power-law (blue) and exponential (red dashed) models shown on a linear scale. The exponential model yields a marginally better group-level fit (R² = 0.992 vs. 0.988; RMSE = 1.92 vs. 2.29). B) Log–log representation of the same data, demonstrating the linearization of the power-law model and enabling direct visual assessment of the decay exponent. Both models are anchored to the observed Cycle 1 mean (x₀ = 59.6%) and have a single free parameter. Error bars indicate ±SEM across subjects. (TIF) [file pcbi.1014572.s013.tif]

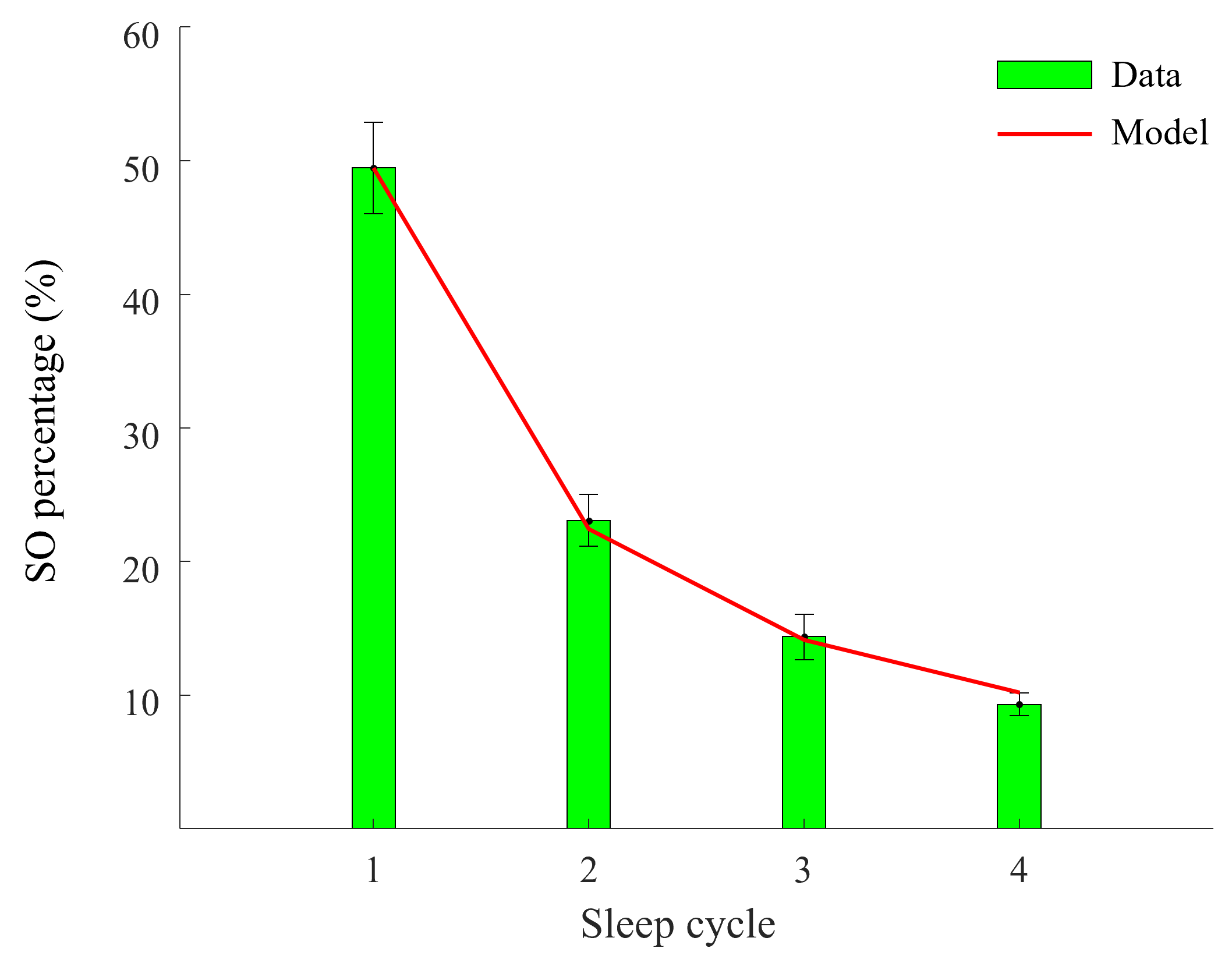

Supplement: S9 Fig — x-axis shows sleep cycle and error bar shows standard error. The decreasing trend in SO percentage across cycles can be described by a power law model with decreasing rate equal to -1.139. The RMSE of the model is 0.0064. (TIF) [file pcbi.1014572.s014.tif]

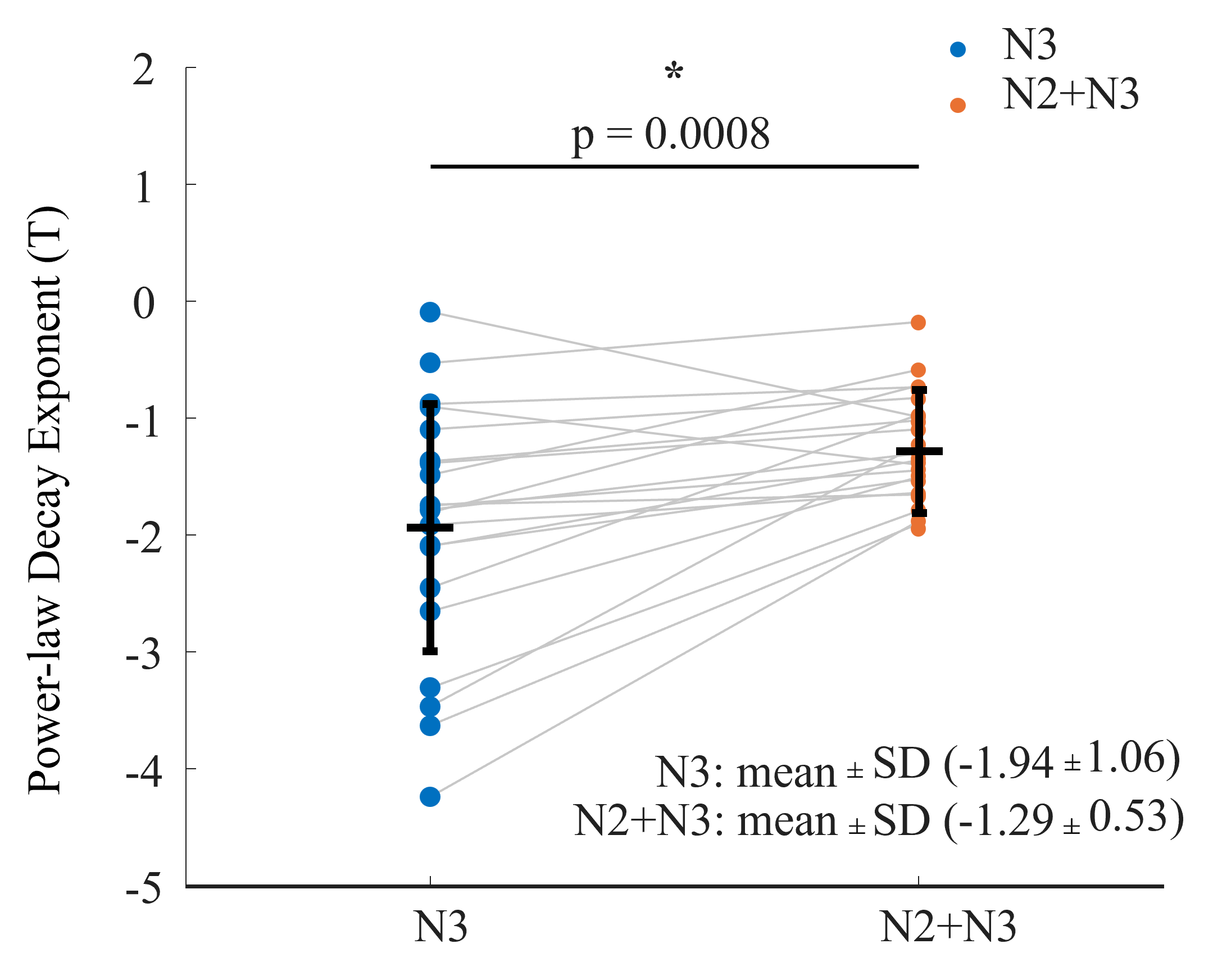

Supplement: S10 Fig — Each point represents one participant’s fitted power-law exponent (T) for either N3-only (blue circles) or combined N2&N3 (red squares). Gray lines connect paired observations within each participant. Black error bars show group mean ± SD. N3 decay was significantly steeper (paired t-test: t(21) = −3.956, p = 0.0008; n = 22 subjects). (TIF) [file pcbi.1014572.s015.tif]

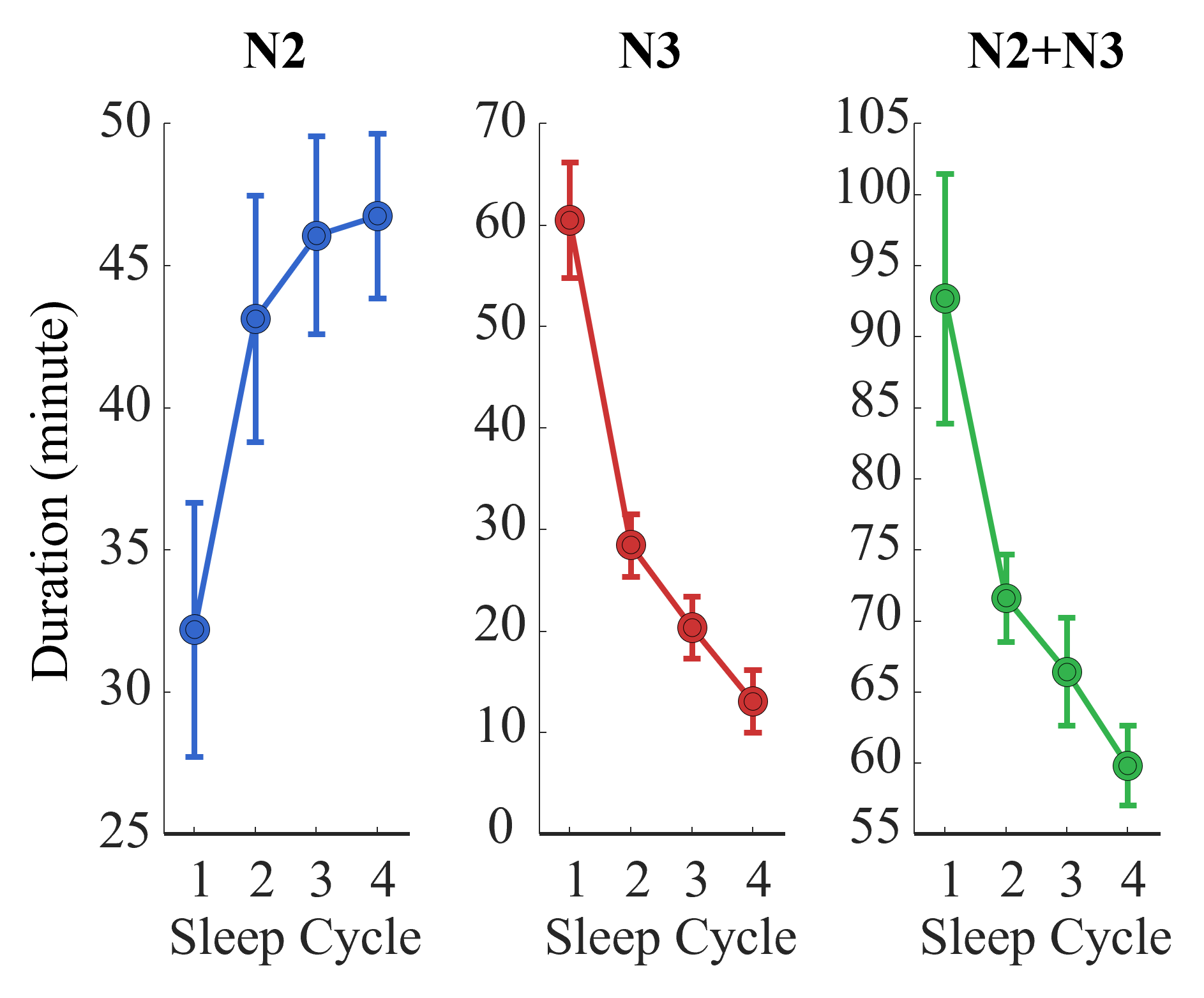

Supplement: S11 Fig — Mean duration (± standard error) of N2, N3, and combined N2 + N3 sleep stages across the first four sleep cycles. N3 duration shows a progressive decline across cycles, while N2 duration increases, reflecting the well-established redistribution of NREM sleep architecture across the night. Total NREM (N2 + N3) duration exhibits a gradual decrease. Each point represents the average across participants, with error bars indicating standard error of the mean. (TIF) [file pcbi.1014572.s016.tif]

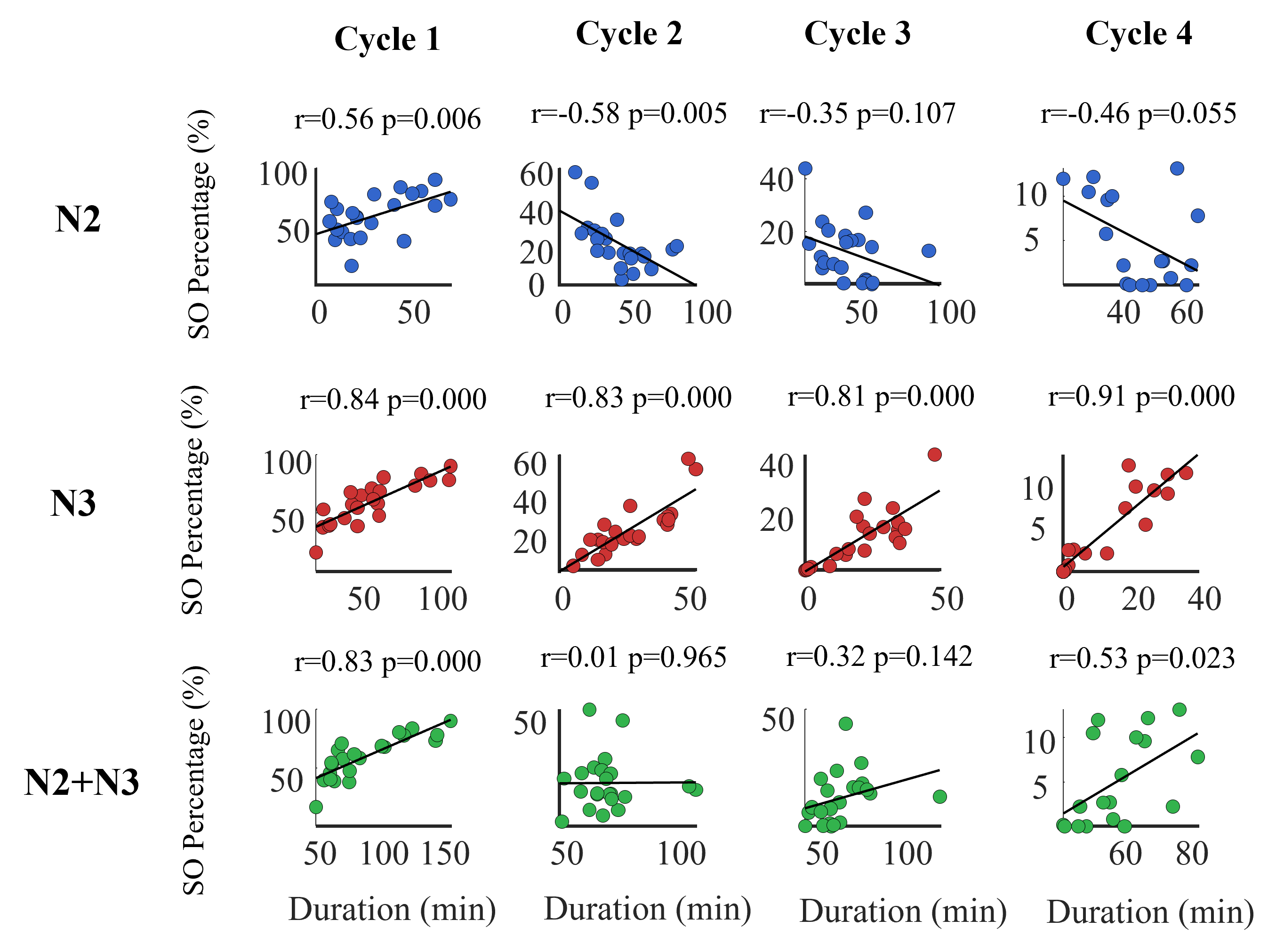

Supplement: S12 Fig — Scatter plots showing the relationship between stage duration (N2, N3, and N2 + N3) and SO percentage across participants for each sleep cycle. Each point represents one participant. N3 duration exhibits a strong positive correlation with SO percentage across all cycles, whereas N2 duration shows weaker and less consistent relationships. Solid lines indicate linear regression fits. (TIF) [file pcbi.1014572.s017.tif]

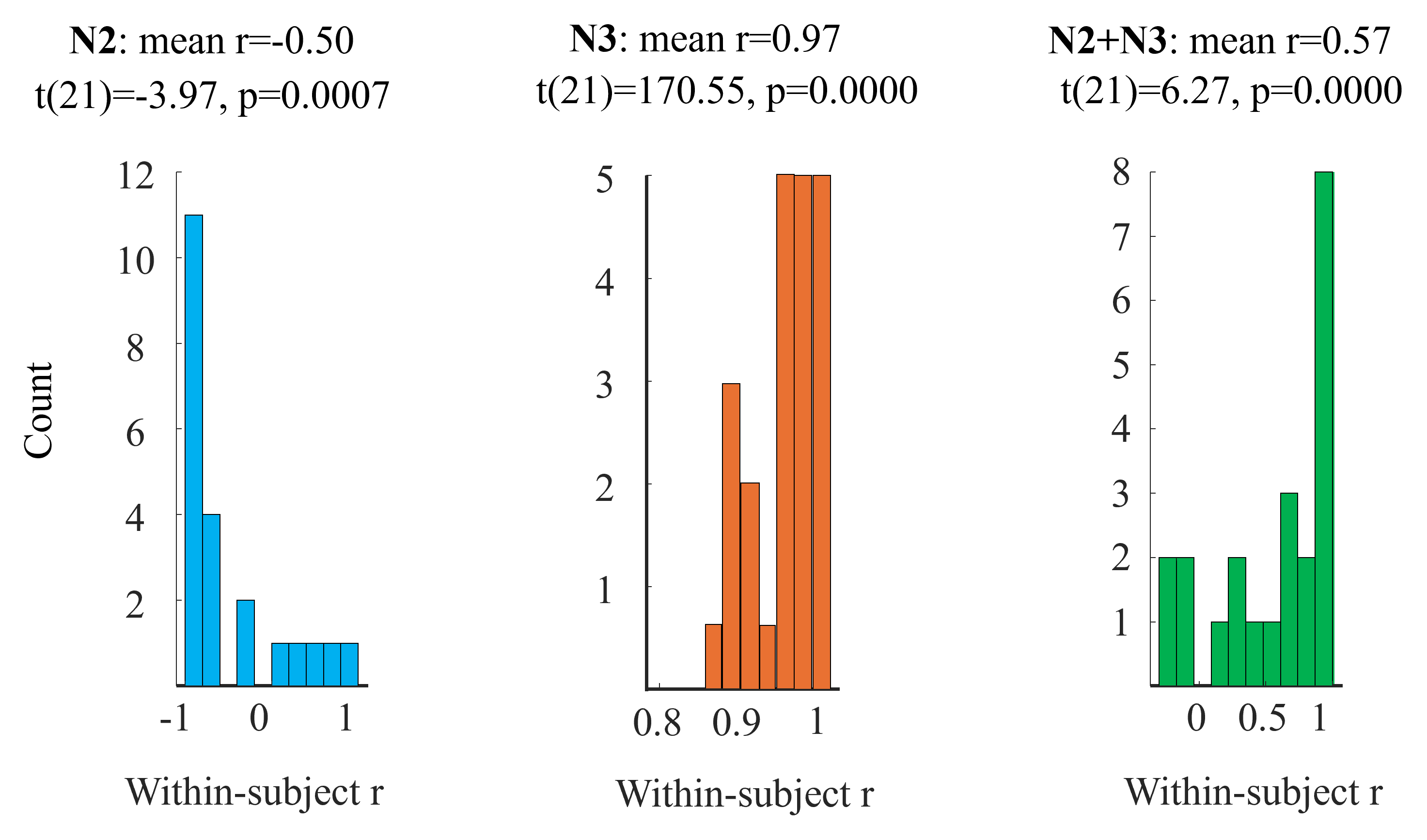

Supplement: S13 Fig — Distribution of within-subject Pearson correlation coefficients (r) quantifying the relationship between stage duration and SO percentage across sleep cycles. Each value represents one participant. N3 duration shows a consistently strong positive relationship with SO percentage across individuals, indicating that reductions in N3 duration closely track decreases in SO expression within subjects. (TIF) [file pcbi.1014572.s018.tif]

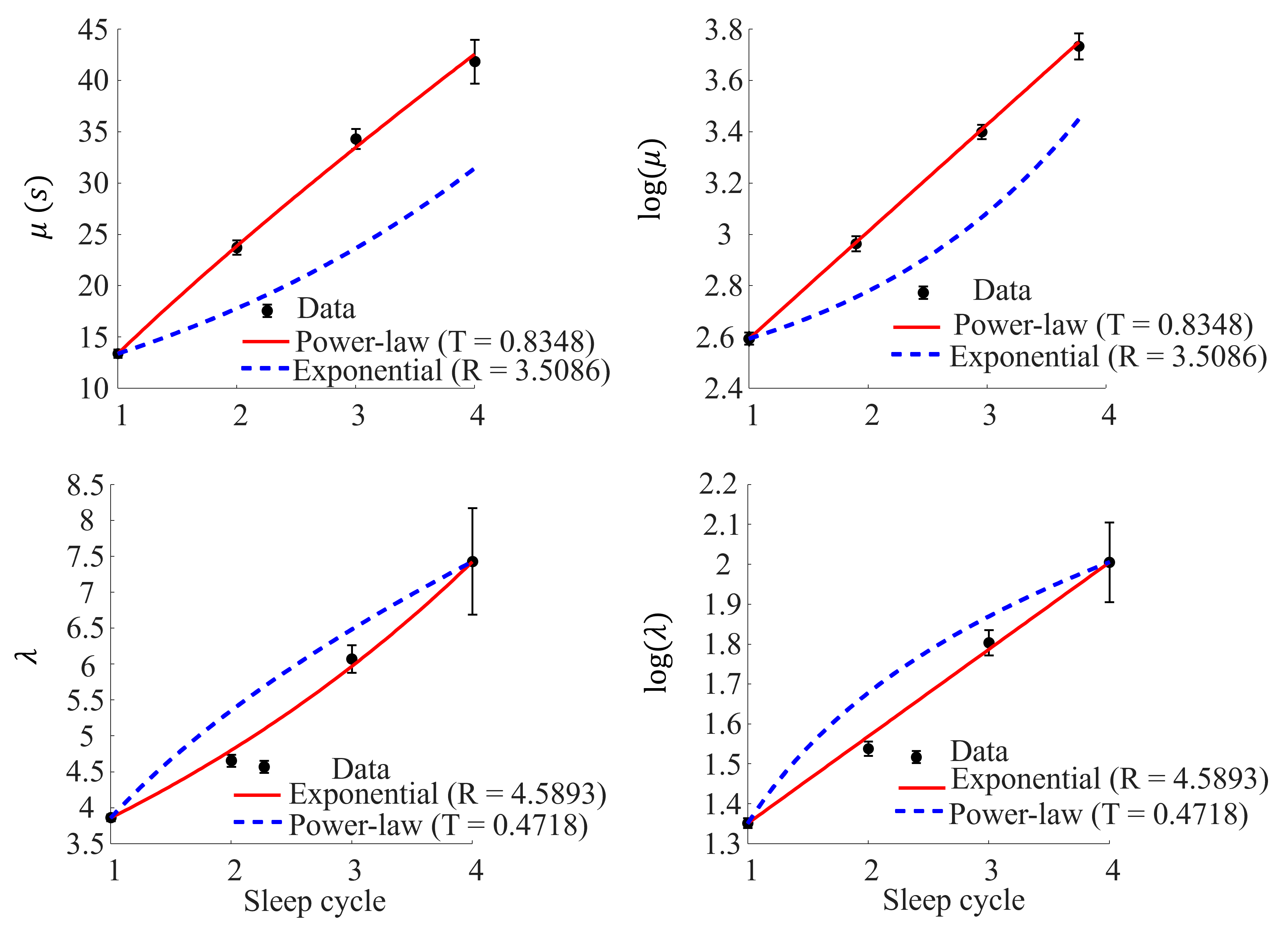

Supplement: S14 Fig — Left column: μ (top) and λ (bottom) plotted on linear scale with power-law (red solid) and exponential (blue dashed) fits overlaid. Right column: log-log scale for μ and log-linear scale for λ, where the preferred model (power-law for μ, exponential for λ) appears as a straight line. Error bars indicate SEM. (TIF) [file pcbi.1014572.s019.tif]

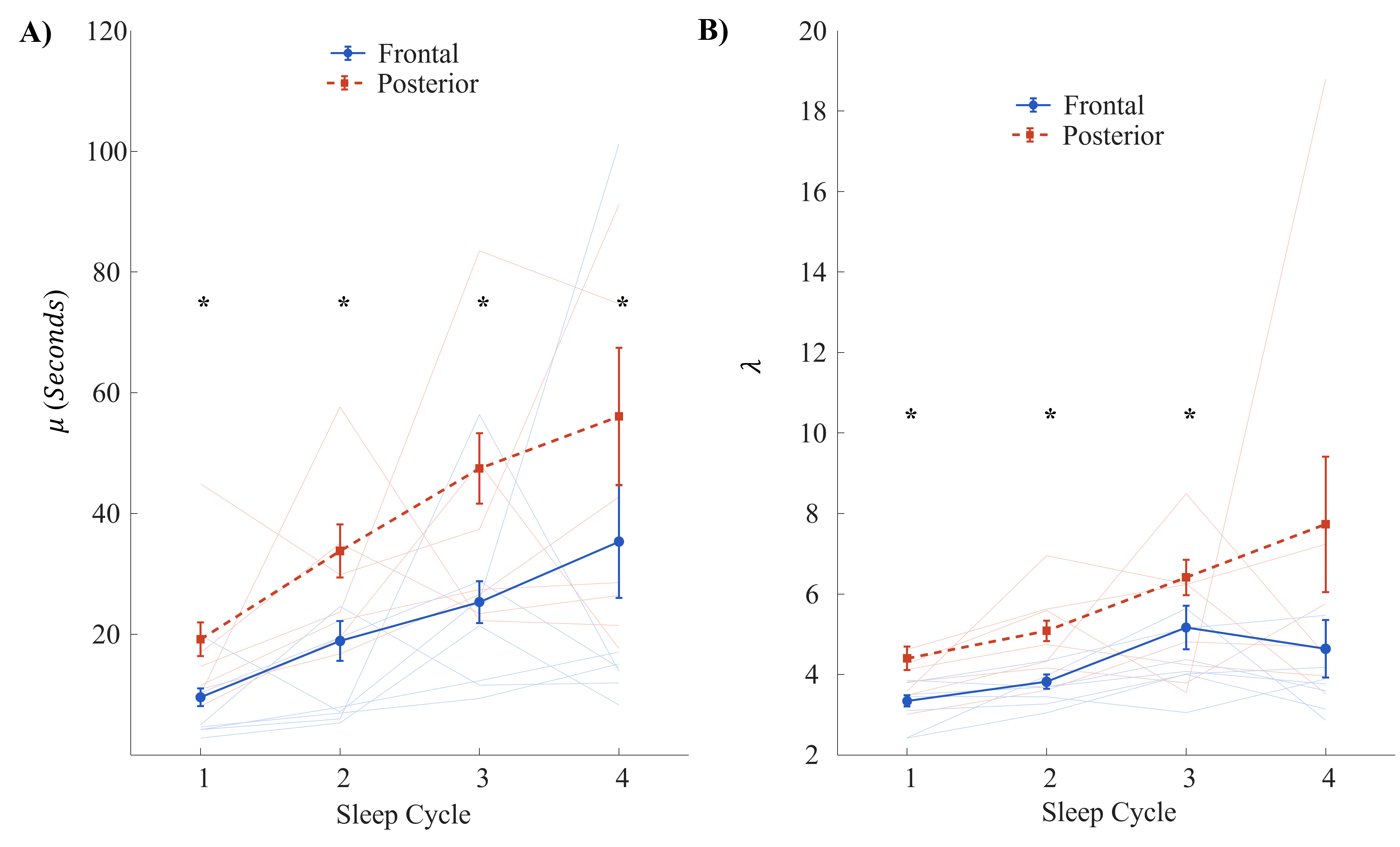

Supplement: S15 Fig — A) Mean μ (inter-SO interval, seconds) and (B) mean λ (shape parameter) across sleep cycles for frontal (FP1, FP2, FPz, F3, F4, Fz, F7, F8) and posterior (P3, P4, P1, P2, P5, P6, Pz, O1, O2, P7, PO3, PO4, POz, P8) electrode groups. Values are shown as mean ± SEM across subjects, with individual subject trajectories displayed in light red (posterior) and blue (frontal). Frontal electrodes exhibit consistently lower μ values, indicating higher SO density, and higher λ values in early cycles, suggesting more regular SO timing. Both regions show similar across-cycle trends. Asterisks indicate significant differences between regions (paired t-test, p < 0.05). (TIF) [file pcbi.1014572.s020.tif]
